# Supplementary material for: Development of type 2 diabetes in women with comorbid gestational diabetes and common mental disorders in the Born in Bradford cohort
Source: BMJ Open. 2022 Mar 14;12(3):e051498. doi: 10.1136/bmjopen-2021-051498 (PMC8921865; doi:10.1136/bmjopen-2021-051498)
Supplement: Supplementary data [file bmjopen-2021-051498supp001.pdf]

Supplementary material S1: indicators of common mental disorders (CMD) from primary care records

Prescriptions

agomelatine, alprazolam, alventa, alventa xl, angilol, ativan, bonilux, bonilux xl, buspirone hydrochloride, chloralbetaine, chloralhydrate, chloral mixture bp2000, cipralex, cipramil, circadin, citalopram, clomipramine, clomipramine hydrochloride, clonezapam, depefex, depefex xl, diazepam, dosulepin, dosulepin hydrochloride, edronax, efexor, efexor xl, escitalopram, feprapax, fluoxetine, flurazepam, fluvoxamine, fluvoxamine maleate, foraven, foraven xl, gamanil, imipramine, imipramine hydrochloride, isocarboxazid, lofepramine, lomont, loprazolam, lorazepam, lormetazepam, lustral, manerix, marplan, melatonin, meprobamate, mianserin, mianserin hydrochloride, mirtazapine, moclobemide, molipaxin, nardil, nitrazepam, optimax, oxactin, oxazepam, parnate, paroxetine, phenelzine, politid, politid xl, propranolol, propranolol hydrochloride, prothiaden, prozac, ranfaxine, ranfaxine xl, reboxetine, seroxat, sertraline, sonata, stilnoct, surmontil, syprol, temazepam, tifaxin, tifaxin xl, tranylcypromine, trazodone, trazodone hydrochloride, trimipramine, tryptophan, valdoxan, venaxx, venaxx xl, venlafaxine, venlafaxine m/r, vensir, vensir xl, welldorm, winfex, winfex xl, zaleplon, zimovane, zispin, zispinsoltab, zolpidem, zolpidem tartrate, zopiclone, allegron, anafranil, anafranil sr, chlordiazepoxide, chlordiazepoxide hydrochloride, clomethiazole, cymbalta, doxepin, duloxetine, rivotril, sinepin, sodium oxybate, triptafen, xyrem, yentreve, amitriptyline, amitriptyline hydrochloride, nortriptyline, promethazine, promethazine hydrochloride

Read codes

Depression:
1B17., 1B19., 1B1U., 2257., 62T1., E112., E1120, E1121, E1122, E1123, E1125, E1126, E112z, E113., E1130, E1131, E1132, E1135, E1136, E1137, E113z, E118., E11y2, E11z0, E11z1, E11zz, E204., E210., E211., E2110, E2112, E2B.., E2B0., E2B1., Eu320, Eu321, Eu322, Eu324, Eu325, Eu326, Eu327, Eu32B, Eu32y, Eu32z, Eu330, Eu331, Eu33y, Eu33z, Eu34., Eu340, Eu34y, Eu34z, Eu3y., Eu3y1, Eu3yy, Eu3z., Eu53., Eu530, X00SO, X00SR, X00SS, X00SU, X00TX, X40Dl, X40Dm, X760u, X7617, X761I, X761J, X761K, X761L, XE0re, XE0uv, XE1Xy, XE1Y0,
XE1Y1, XE1YC, XE1ZY, XE1Za, XE1Zb, XE1Zc, XE1Zd, XE1Zf, XE1Zg, XE1Zh, XE1Zi, XE1aY, XE1ae, XM0Ar, XM0CR, XM1GC, XSEGJ, XSGok, XSGol, XSGom, Xa02E, Xa0wV, Xa110, Xa17z, Xa1eL, Xa9E0, Xa9J0, Xa9K0, XaCHr, XaCHs, XaCIs, XaCIt, XaCIu, XaImU, XaJWh, XaKUk, XaPKm, XaPOv, XaX0C, XaY2C, XaAyL, XaB5v, XaB95, XaB9J

Anxiety:
1B13., 1B1V., 2258., 225J., E0300, E0310, E200., E2000, E2001, E2002, E2004, E2005, E200z, E201., E2010, E2011, E2012, E2013, E2014, E2015, E2016, E2017, E2018, E201A, E201B, E201C, E201z, E202., E2020, E2021, E2022, E2023, E2024, E2025, E2026, E2027, E2028, E2029, E202A, E202B, E202C, E202D, E202E, E202z, E203., E2030, E2031, E203z, E205., E207., E20y., E20y0, E20y1, E20y2, E20y3, E20yz, E20z., E28.., E280., E281., E282.,
E283., E2830, E2831, E283z, E284., E28z., Eu40., Eu400, Eu401, Eu402, Eu40y, Eu40z, Eu41., Eu410, Eu411, Eu41y, Eu41z, Eu42., Eu420, Eu421, Eu422, Eu42y, Eu42z, Eu515, Eu51y, Eu51z, Ub1T9, X00Sc, X00Sf, X761N, XE0rb, XE1Y7, XE1YA, XE1Ym, XE1Yn, XE1Zj, XE1aW, XE1bo, XM1MZ, Xa0XG, Xa0XH, Xa0XI, Xa0XJ, Xa0XK, Xa0XM, Xa0XN, Xa0XO, Xa0XP, Xa0XQ, Xa0XR, Xa0XX, Xa0XY, Xa0Xd, Xa18j, Xa18v, Xa19B, Xa3Xk, Xa3Ys, Xa7kB, XaEFB, XaP8d, XaX55, XaX56, XaX58

Comorbid depression and anxiety:
E2003, Eu412, Eu413, X00Sb

CMD treatment or referral for CMD treatment:
6655., 6659., 66590, 6779., 6G00., 8BK0., 8BM0., 8CQ.., 8CR7., 8F85., 8G..., 8G1.., 8G10., 8G100, 8G11., 8G12., 8G120, 8G121, 8G2.., 8G21., 8G2Z., 8G4.., 8G43., 8G4Z., 8G5.., 8G51., 8G5Z., 8G6.., 8G6Z., 8G7.., 8G7Z., 8G9.., 8G91., 8G9Z., 8HlB., 8HVO., 8H23., 8H230, 8H34., 8H38., 8H49., 8H7A., 8H7B., 8H7T., 8H7Z., 8HHp., 8HHq., 8HJ3., 8HK9., 8HkK., 8HM9., 9HZ.., 9N0T., 9N1M., 9N2B., 9N6h., 9NJ1., 9NJR., 9NJT., 9Ol.., Ub0qs, X71Ec, X71bp, X79sL, XE0iL, XE1Sa, XE1Sb, XSBbs, Xa8IB, Xa8IG, Xa8IJ, Xa8IP, Xa8IR, Xa8If, Xa8Ig, Xa8Ih, Xa8Ii, Xa8Ij, Xa8Ik, Xa8Is, Xa8It, Xa8Iu, Xa8Ix, Xa8J0, XaA8Z, XaA8c, XaA8d, XaA8u, XaA8v, XaA9W, XaA9g, XaABP, XaABQ, XaAKy, XaAMj, XaAMz, XaAOd, XaAOe, XaAOf, XaAOg, XaAOh, XaAQi, XaAQo, XaAS4, XaAU5, XaAUA, XaAXe, XaAZl, XaAbC, XaAbH, XaAdM, XaAel, XaAem, XaAen, XaAfJ, XaAh4, XaAiE, XaAiI, XaAkB, XaAkI, XaAkU, XaAnb, XaBHK, XaBIg, XaBJb, XaBJc, XaBT1, XaBTD, XaBtN, XaBvV, XaBvW, XaBvX, XaCFD, XaECG, XaEVq, XaI8j, XaINQ, XaINy, XaIOf, XaIOg, XaIOh, XaIOi, XaIOj, XaIOk, XaIOl, XaIOn, XaIOp, XaIOq, XaIOs, XaIOu, XaIOv, XaIOy, XaIOz, XaIP0, XaIP1, XaIP2, XaIP3, XaIPw, XaISp, XaISv, XaISw, XaISy, XaIT1, XaIT2, XaIT3, XaIT4, XaIT5, XaIT6, XaIT7, XaIT8, XaITA, XaITG, XaITH, XaITI, XaIUv, XaIUx, XaIUy, XaIUz, XaIV0, XaIV1, XaIV2, XaIV3, XaIV4, XaIV5, XaIV6, XaIW3, XaIW4, XaIW5, XaIW6, XaIWD, XaIWM, XaIWN, XaIWR, XaIWS, XaIWT, XaIWU, XaIWV, XaIWW, XaIWX, XaIWY, XaIWZ, XaIWa, XaIWb, XaIWx, XaIWy, XaIWz, XaIX0, XaIXS, XaIXT, XaIXU, XaIXV, XaIXW, XaIXX, XaIXY, XaIXZ, XaIXa, XaIXb, XaIXh, XaIXi, XaIXk, XaIXl, XaIXm, XaIXn, XaIXo, XaIXp, XaIXq, XaIXs, XaIXt, XaIXu, XaIYN, XaIkd, XaIkg, XaIku, XaIm4, XaIpA, XaItc, XaItx, XaIuR, XaIvk, XaIvp, XaIvq, XaIyU, XaJ4V, XaJ4w, XaJ4x, XaJOA, XaJON, XaJPu, XaJPz, XaJQ1, XaJQD, XaJQE, XaJQF, XaJQG, XaJQH, XaJQI, XaJQJ, XaJQR, XaJQS, XaJQT, XaJQU, XaJQV, XaJQW, XaJQX, XaJQY, XaJQZ, XaJRr, XaJWg, XaJr3, XaK1f, XaK5q, XaK5r, XaK6K, XaK70, XaK71, XaKAx,
XaKEz, XaKGq, XaKbb, XaL03, XaL0o, XaL0p, XaL0q, XaL0r, XaL0s, XaL0t, XaL0u, XaL0v, XaL0w, XaL2L, XaLBl, XaLCP, XaLCQ, XaLFL, XaLFk, XaLNF, XaLQw, XaLnp, XaLnq, XaLnr, XaLst, XaLsu, XaLsv, XaM2K, XaM7s, XaMGz, XaMJ8, XaMhM, XaN3a, XaN4b, XaN4c, XaN4d, XaN4e, XaN4f, XaN4g, XaNPL, XaNTc, XaONq, XaOOT, XaObo, XaOxM, XaP6T, XaP7x, XaPRF, XaPTT, XaPTU, XaPlZ, XaPvy, XaPvw, XaQBz, XaQC0, XaQWJ, XaQvz, XaR4n, XaR4s, XaR5D, XaWzW, XaX04, XaXEJ, XaXH8, XaXHm, XaXe3, XaXiH, XaXl2, XaY6o, XaY7i, XaYgS, XaZIW, XaZcf, ZV663, ZV673, ZV69., ZV690, ZV691, ZV692, ZV6D., ZV701, ZV702

CMD-related follow-up:
665.., 6654., 6658., 66580, 665A., 665A0, 665Z., 8A2.., 8A21., 8A2Z., 9H90., 9H91., 9H92., 9HA0., 9Ov.., 9Ov0., 9Ov1., 9Ov2., 9Ov3., 9Ov4., X74WN, XaJuG, XaJuK, XaJuT, XaJuV, XaJuW, XaK6d, XaK6e, XaK6f, XaK9p, XaKAK, XaLIb, XaMGL, XaMGN, XaMGO, XaMGP, XaMGQ, XaMGR, XaR9y, XaZ2p

CMD History:
146.., 1465., 1466., 1467., 146A., 146G., 146Z., 9HA1., Eu334, Xa41K, XaJWi, XaLG., ZV111

Timing of preconception CMD

Women and all of their pregnancies were coded as affected by preconception CMD if there was attached to that woman any prescription or Read code for depression, anxiety, comorbid depression and anxiety, treatment of, follow up for or history of anxiety or depression dated prior to the date of conception of the first pregnancy; the first pregnancy for each woman may or may not have been a BiB pregnancy.
380 of 13,539 pregnancies in the sample had a missing date of conception due to unknown gestational age or date of delivery. In women with a pregnancy with a missing date of conception whose first pregnancy was a BiB pregnancy, if the date attached to the Read code or prescription was prior to 1 July 2006 (the earliest possible date of conception based on first recruitment into the

study), these women and their pregnancies were recorded as affected by preconception CMD. Otherwise the woman was recorded as missing information about preconception CMD (it would seem erroneous for such women to take the default of no preconception CMD which was used for women in which no Read or prescription codes were recorded).

Women with recorded parity exceeding zero (i.e. multiparity) or missing parity in their first BiB pregnancy (i.e. births occurring prior to entry into BiB with unknown date of conception) and with a Read or prescription code, would also have had to be coded as missing, i.e. they may have had preconception CMD, but the date of conception of their first birth is not known. However, extraction of data from maternity records pertaining to pre-BiB pregnancies provided years of pregnancies prior to the BiB study for 5611 women. If the date of the primary care code preceded the earliest pregnancy date for that mother (only year was available so the date of pregnancy was elected to be the earliest possible at 1 January), she was coded as affected by preconception CMD. For multiparous women without this information available, she was recorded as missing information about preconception CMD following the same rationale as the paragraph above.

Timing of antenatal CMD

Women were coded as experiencing antenatal CMD if there was a prescription or Read code for CMD in the mother's records between the estimated date of conception and date of delivery.

Supplementary material S2: indicators of severe mental illness (SMI) from primary care records

Women with a Read code or medication prescription for SMI at any point in their lifetime were coded as having SMI.

Prescriptions

abilify, acuphase, amisulpride, anquil, aripiprazole, asenapine, benperidol, camcolit, clopixol, clopixol, clozapine, clozaril, denzapine, depixol, depixol conc, depixol low volume, dolmatil, fluanxol, flupentixol, flupentixol decanoate, fluphenazine decanoate, haldol decanoate, invega, li-liquid, liskonum, lithium carbonate, lithium citrate, modecate, modecate concentrate, olanzapine, olanzapine embonate, orap, paliperidone, pericyazine, pimozide, piportil depot, pipotiazine, palmitate, priadel, promazine, promazine hydrochloride, quetiapine, risperdal, risperdal consta, risperidone, seroquel, seroquel xl, solian, sulpiride, sulpor, sycrest, xeplion, zaponex, zuclopenthixol, zuclopenthixol acetate, zuclopenthixol decanoate, zypadhera, haldol decanoate, Zyprexa, carbamazepine, chlorpromazine, chlorpromazine hydrochloride , convulex, depakote, dozic, fentazin, haldol, haloperidol, largactil, levomepromazine, nozinan, perphenazine, serenace, stelazine, tegretol trifluoperazine, valproic acid

Read codes

1B1b., 225E., 225F., 6656., 6657., 665B., 665C., 665D., 665E., 665F., 665G., 665H., 665J., 665K., 8HHs., 9H1.., 9H11., 9H12., 9H13., 9H14., 9H1Z., 9H2.., 9H21., 9H22., 9H23., 9H24., 9H25., 9H2Z., 9H3.., 9H31., 9H32., 9H33., 9H34., 9H3Z., 9H4.., 9H41., 9H42., 9H43., 9H44., 9H45., 9H4Z., 9H5.., 9H51., 9H52., 9H53., 9H54., 9H55., 9H5Z., 9H7.., 9H8.., 9Ol6., 9Ol7., E10.., E100., E1000, E1001, E1002, E1003, E1004, E1005, E100z, E101., E1010, E1011,
E1012, E1013, E1014, E1015, E101z, E102., E1020, E1021, E1022, E1023, E1024, E1025, E102z, E103., E1030, E1031, E1032, E1033, E1034, E1035, E103z, E104., E110., E1100, E1101, E1102, E1103, E1104, E1105, E1106, E110z, E111., E1110, E1111, E1112, E1113, E1114, E1115, E1116, E111z, E1124, E1133, E1134, E114., E1140, E1141, E1142, E1143, E1144, E1145, E1146, E114z, E115., E1150, E1151, E1152, E1153, E1154, E1155, E1156,
E115z, E116., E1160, E1161, E1162, E1163, E1164, E1165, E1166, E116z, E117., E1170, E1171, E1172, E1173, E1174, E1175, E1176, E117z, E11y., E11y0, E11y1, E11y3, E11yz, E11z., E12.., E120., E121., E122., E123., E12y., E12y0, E12yz, E12z., E13.., E130., E131., E132., E133., E134., E135., E13y., E13y0, E13y1, E13yz, E13z., E14.., E141., E1411, E141z, E14y., E14y1, E14yz, E14z., E1y.., E1z.., E21.., E2111, E2112, E2113, E211z, E212., E2120,
E2121, E2122, E212z, E213., E214., E2140, E2141, E214z, E215., E2150, E2151, E2152, E2153, E215z, E216., E217., E21y., E21y1, E21y2, E21y3, E21y4, E21y5, E21y6, E21y7, E21yz, E21z., Eu1.., Eu10., Eu100, Eu101, Eu102, Eu103, Eu104, Eu105, Eu106, Eu107, Eu108, Eu10y, Eu10z, Eu11., Eu110, Eu111, Eu112, Eu113, Eu114, Eu115, Eu116, Eu117, Eu11y, Eu11z, Eu12., Eu120, Eu121, Eu122, Eu123, Eu124, Eu125, Eu126, Eu127, Eu12y, Eu12z, Eu13., Eu130, Eu131, Eu132, Eu133, Eu134, Eu135, Eu136, Eu137, Eu13y, Eu13z, Eu14., Eu140, Eu141, Eu142, Eu143, Eu144, Eu145, Eu146, Eu147, Eu14y, Eu14z, Eu15., Eu150, Eu151, Eu152, Eu153, Eu154, Eu155, Eu156, Eu157, Eu15y, Eu15z, Eu16., Eu160, Eu161, Eu162, Eu163, Eu164, Eu165, Eu166, Eu167, Eu16y, Eu16z, Eu17., Eu170, Eu171, Eu172, Eu173, Eu174, Eu175, Eu176, Eu177, Eu17y, Eu17z, Eu18., Eu180, Eu181, Eu182, Eu183, Eu184, Eu185, Eu186, Eu187, Eu18y, Eu18z, Eu19., Eu190, Eu191, Eu192, Eu193, Eu194, Eu195, Eu196, Eu197, Eu19y, Eu19z, Eu1A., Eu1A0, Eu1A1, Eu1A2, Eu1A3, Eu1A4, Eu1A5, Eu1A6, Eu1A7, Eu1Ay, Eu1Az, Eu2.., Eu20., Eu200, Eu201, Eu202, Eu203, Eu204, Eu205, Eu206,

Eu20y, Eu20z, Eu21., Eu22., Eu220, Eu221, Eu222, Eu223, Eu22y, Eu22z, Eu23., Eu230, Eu231, Eu232, Eu233, Eu23y, Eu23z, Eu24., Eu25., Eu250, Eu251, Eu252, Eu25y, Eu25z, Eu26., Eu2y., Eu2z., Eu30., Eu300, Eu301, Eu302, Eu30y, Eu30z, Eu31., Eu310, Eu311, Eu312, Eu313, Eu314, Eu315, Eu316, Eu317, Eu318, Eu319, Eu31y, Eu31z, Eu323, Eu328, Eu329, Eu32A, Eu333, Eu341, Eu3z., Eu44., Eu440, Eu441, Eu442, Eu443, Eu444, Eu445, Eu446, Eu447, Eu44y, Eu44z, Eu45., Eu450, Eu451, Eu452, Eu453, Eu454, Eu455, Eu45y, Eu45z, Eu46., Eu460, Eu461, Eu46y, Eu46z, Eu5.., Eu531, Eu54., Eu55., Eu5z., Eu6.., Eu61., Eu60., Eu601, Eu602, Eu603, Eu604, Eu605, Eu606, Eu607, Eu608, Eu60y, Eu60z, Eu62., Eu620, Eu621, Eu62y, Eu62z, Ua1WW, Ub1T7, X73gl, X73gm, X73gn, X73go, X75yp, X75yv, X75yw, X75z5, X75z7, X75zA, X75zC, X75zE, X761M, X79ul, XE1Xt, XE1Xw, XE1Xx, XE1Y2, XE1Y3, XE1Y4, XE1Y5, XE1Y6, XE1YF, XE1YG, XE1YH, XE1YI, XE1YJ, XE1YK, XE1YL, XE1YM, XE1ZM, XE1ZN, XE1ZO, XE1ZP, XE1ZQ, XE1ZR, XE1ZU, XE1ZX, XE1ZZ, XE1Ze, XE1Zy, XE1aM, XE1aO, XE1aQ, XE1aS, XE1aU, XE1ag, XE1am, XE1gG, XE1ic, XE1ji, XE2RN, XE2b6, XE2b8, XE2uT, XE2un, XE2v2, XM1GG, XM1Yd, XSGon, Xa1aD, Xa1hV, Xa3WO, Xa3Xd, Xa3Xf, Xa3Xg,
Xa3Y9, Xa3aC, Xa3aF, Xa3aL, Xa3aP, Xa3aU, Xa3aV, Xa3aW, Xa3aX, Xa3at, Xa4HV, Xa4Ha, Xa8Nf, Xa8O2, Xa8OA, Xa8OE, Xa8Og, Xa8Oh, Xa8Oi, Xa8Pk, Xa8Qw, Xa8Qx, Xa8Qy, Xa8Qz, Xa9Dh, Xa9Di, Xa9Dk, Xa9Dm, Xa9Do, Xa9Dr, Xa9Ds, Xa9Dt, Xa9Du, Xa9Dv, Xa9Dw, Xa9Dx, Xa9Dy, Xa9Dz, Xa9E1, Xa9E2, Xa9EC, Xa9EE, Xa9EF, Xa9EG, Xa9EI, Xa9EM, Xa9EP, Xa9EQ, Xa9ER, Xa9EV, Xa9EW, Xa9EX, Xa9EY, Xa9EZ, Xa9Ea, Xa9Eb, Xa9Ec, Xa9Ed, Xa9Ee, Xa9Ef, Xa9Eg, Xa9Ei, Xa9Ej, Xa9Ek, Xa9El, Xa9Em, Xa9Eo, Xa9Ep, Xa9Eq, Xa9Er, Xa9Es, Xa9Et, Xa9Eu,
Xa9Ev, Xa9GR, Xa9GU, Xa9GV, Xa9GX, Xa9GZ, Xa9Gc, Xa9IW, Xa9IX, Xa9IY, Xa9IZ, Xa9Ib, Xa9Ic, Xa9Id, Xa9Ie, Xa9If, Xa9Ig, Xa9Ih, Xa9Ii, Xa9Ij, Xa9Ik, Xa9Il, Xa9Im, Xa9In, Xa9Ip, Xa9Iq, Xa9Is, Xa9Iw, Xa9Iy, Xa9J3, Xa9J4, Xa9J5, Xa9J6, Xa9JA, Xa9JC, Xa9JE, Xa9JG, Xa9JH, Xa9JJ, Xa9JK, Xa9JR, Xa9Ja, Xa9Jb, Xa9Jd, Xa9Jf, Xa9Jh, Xa9Jj, Xa9Jl, Xa9Jn, Xa9Jp, Xa9Jq, Xa9Jr, Xa9Js, Xa9Jt, Xa9Jz, Xa9K2, Xa9K3, Xa9K4, Xa9K5, XaA6j, XaA6x, XaA9j, XaA9r, XaA9s, XaBHL, XaBHM, XaBHN, XaBHO, XaBYV, XaBYW, XaBYX, XaBYY, XaBYZ, XaBhM, XaCHo, XaIOm, XaIWE, XaIWF, XaIWG, XaIWH, XaIWI, XaIWJ, XaIWK, XaIWL, XaIXj, XaJQO, XaKUl, XaKUm, XaL19, XaLIa, XaMwc, XaMwd, XaMwe, XaNlN, XaPYK, XaPYL, XaX51, XaX52, XaX53, XaX54, XaY1Y

Supplementary material S3: Complete case analysis of Cox regression for Type 2 diabetes in women with vs without an indicator of antenatal CMD (N=750 women with GDM)

Complete case analysis of Cox regression for Type 2 diabetes in women with vs without an indicator of antenatal CMD (N=750 women with GDM)

	Type 2 diabetes		
**Unadjusted**	**HR**	**(95% CI)**	**p**
**Antenatal CMD**			
Reference category= no indicator			
Antenatal CMD indicator	1.12	(0.66, 1.92)	0.668
**Adjusted***	**HR**	**(95% CI)**	**p**
**Antenatal CMD**			
Reference category= no indicator			
Antenatal CMD indicator	1.12	(0.65, 1.94)	0.685

**adjusted for maternal age, education, ethnicity, smoking and preconception CMD*
