# Supplementary material for: Development of type 2 diabetes in women with comorbid gestational diabetes and common mental disorders in the Born in Bradford cohort
Source: BMJ Open. 2022 Mar 14;12(3):e051498. doi: 10.1136/bmjopen-2021-051498 (PMC8921865; doi:10.1136/bmjopen-2021-051498)
Supplement: Author's manuscript [file bmjopen-2021-051498.draft_revisions.pdf]

BMJ Open

BMJ Open is committed to open peer review. As part of this commitment we make the peer review history of every article we publish publicly available.

When an article is published we post the peer reviewers' comments and the authors' responses online. We also post the versions of the paper that were used during peer review. These are the versions that the peer review comments apply to.

The versions of the paper that follow are the versions that were submitted during the peer review process. They are not the versions of record or the final published versions. They should not be cited or distributed as the published version of this manuscript.

BMJ Open is an open access journal and the full, final, typeset and author-corrected version of record of the manuscript is available on our site with no access controls, subscription charges or pay-per-view fees (http://bmjopen.bmj.com).

If you have any questions on BMJ Open's open peer review process please email info.bmjopen@bmj.com

BMJ Open

Development of Type 2 diabetes in women with co-morbid gestational diabetes and common mental disorders in the Born in Bradford cohort

Journal:	*BMJ Open*
Manuscript ID	bmjopen-2021-051498
Article Type:	Original research
Date Submitted by the Author:	20-Mar-2021
Complete List of Authors:	Wilson, Claire; King's College London, Section of Women's Mental Health; South London and Maudsley NHS Foundation Trust,<br>Santorelli, Gillian; Bradford Teaching Hospitals NHS Foundation Trust<br>Reynolds, Rebecca; University of Edinburgh,<br>Simonoff, Emily; King's College London, Institute of Psychiatry, Child & Adolescent Psychiatry<br>Howard, Louise; institute of psychiatry, health service and population research<br>Ismail, Khalida; Institute of Psychiatry, Kings College London
Keywords:	General diabetes < DIABETES & ENDOCRINOLOGY, EPIDEMIOLOGY, Diabetes in pregnancy < DIABETES & ENDOCRINOLOGY, Maternal medicine < OBSTETRICS, Depression & mood disorders < PSYCHIATRY, PUBLIC HEALTH

SCHOLARONE™
Manuscripts

I, the Submitting Author has the right to grant and does grant on behalf of all authors of the Work (as defined in the below author licence), an exclusive licence and/or a non-exclusive licence for contributions from authors who are: i) UK Crown employees; ii) where BMJ has agreed a CC-BY licence shall apply, and/or iii) in accordance with the terms applicable for US Federal Government officers or employees acting as part of their official duties; on a worldwide, perpetual, irrevocable, royalty-free basis to BMJ Publishing Group Ltd ("BMJ") its licensees and where the relevant Journal is co-owned by BMJ to the co-owners of the Journal, to publish the Work in this journal and any other BMJ products and to exploit all rights, as set out in our licence.

The Submitting Author accepts and understands that any supply made under these terms is made by BMJ to the Submitting Author unless you are acting as an employee on behalf of your employer or a postgraduate student of an affiliated institution which is paying any applicable article publishing charge ("APC") for Open Access articles. Where the Submitting Author wishes to make the Work available on an Open Access basis (and intends to pay the relevant APC), the terms of reuse of such Open Access shall be governed by a Creative Commons licence – details of these licences and which Creative Commons licence will apply to this Work are set out in our licence referred to above.

Other than as permitted in any relevant BMJ Author's Self Archiving Policies, I confirm this Work has not been accepted for publication elsewhere, is not being considered for publication elsewhere and does not duplicate material already published. I confirm all authors consent to publication of this Work and authorise the granting of this licence.

For peer review only

20/03/2021

Dear editor,

Re: Development of Type 2 diabetes in women with co-morbid gestational diabetes and common mental disorders in the Born in Bradford cohort

Thank you for considering our manuscript for publication. It is one of the largest studies in the UK to date investigating the risk of Type 2 diabetes following gestational diabetes and in this manuscript we specifically aimed to compare the risk in women exposed to the common mental disorders of anxiety and depression during pregnancy versus those unexposed.

In our manuscript, we investigate our novel hypothesis on the impact of mental health during pregnancy on subsequent risk of Type 2 diabetes in women with gestational diabetes. While we found no evidence for an increased risk in women with common mental disorders, as the first study to investigate this research question, we believe that this provides a valuable addition to the research literature. Its findings are also of importance to clinicians involved in the ongoing care of women with gestational diabetes in the postnatal period, as the findings could provide reassurance to women experiencing these co-morbidities (of gestational diabetes and common mental disorders) in pregnancy.

Thank you for your consideration,

Dr Claire Wilson and co-authors

For peer review only - http://bmjopen.bmj.com/site/about/guidelines.xhtml

Development of Type 2 diabetes in women with co-morbid gestational diabetes and common mental disorders in the Born in Bradford cohort

Claire A Wilson, Section of Women's Mental Health, PO31 King's College London, De Crespigny Park, London, SE5 8AF and South London and Maudsley NHS Foundation Trust, UK

Corresponding author e-mail: claire.1.wilson@kcl.ac.uk; tel: 07729 324100, ORCID ID: 0000-0003-2169-5115

Gillian Santorelli, Born in Bradford, Bradford Teaching Hospitals NHS Foundation Trust, Bradford, UK

Rebecca M Reynolds, Centre for Cardiovascular Science, University of Edinburgh, UK

Emily Simonoff, Department of Child and Adolescent Psychiatry, King's College London and South London and Maudsley NHS Foundation Trust, UK

Louise M Howard*, Section of Women's Mental Health, King's College London and South London and Maudsley NHS Foundation Trust, UK

Khalida Ismail*, Department of Psychological Medicine, King's College London and South London and Maudsley NHS Foundation Trust, UK

*joint senior authors

Author contributions:

CAW conceived the research question and designed the study analysis plan. All authors approved the data analysis plan. GS extracted the data from primary care records. CAW analysed the data and wrote the first draft of the manuscript. All authors critically revised this draft and have approved the final version.

Funding:

Born in Bradford receives core infrastructure funding from the Wellcome Trust (WT101597M) and a joint grant from the UK Medical Research Council (MRC) and Economic and Social Science Research Council (ESRC) (MR/N024397/1). CAW carried out this work as part of a UK Medical Research Council (MRC) funded Clinical Research Training Fellowship (MR/P019293/1). RMR acknowledges the support of Tommy's and the British Heart Foundation (RE/18/5/34216). KI and LMH receive salary support from the National Institute for Health Research (NIHR) Biomedical Research Centre at South London and Maudsley NHS Foundation Trust and King's College London. LMH also receives salary support from the NIHR South London Applied Research Collaboration (ARC).

Competing interests:

None to declare.

Patient consent for publication:

Not applicable.

Ethics approval:

Ethical approval for data collection in BiB was granted by Bradford Research Ethics Committee (Ref 07/H1302/112).

Data availability statement:

Information about access to data and collaborations available at https://borninbradford.nhs.uk/research/how-to-access-data/

For peer review only

Abstract

Objectives: to compare, in a population of women with gestational diabetes, the time to diagnosis of Type 2 diabetes in those with and without common mental disorder (depression and/or anxiety) during pregnancy.

Design and setting: prospective study of the Born in Bradford cohort in Bradford, UK.

Participants: 909 women diagnosed with gestational diabetes between 2007 and 2010, with linkage to their primary care records until 2017. The exposed population were women with an indicator of common mental disorder during pregnancy in primary care records. The unexposed were those without an indicator.

Outcome measures: time to diagnosis of Type 2 diabetes as indicated by a diagnosis in primary care records.

Analysis: time to event analysis using Cox regression was employed. Multiple imputation by chained equations was implemented to handle missing data. Models were adjusted for maternal age, ethnicity, education, preconception common mental disorders and tobacco smoking during pregnancy.

Results: 165 women (18%) were diagnosed with Type 2 diabetes over a follow-up period of around 10 years. There was no evidence of an effect of antenatal CMD on the development of Type 2 diabetes following GDM (adjusted HR 0.95; 95% CI 0.57, 1.57).

Conclusions: women with common mental disorders were not at an increased risk of Type 2 diabetes following gestational diabetes. This is reassuring for women with these co-morbidities but requires replication in other study populations.

Strengths and limitations of this study

- First study to investigate the impact of mental disorder during pregnancy in women with gestational diabetes on subsequent Type 2 diabetes risk.
- Large cohort study of a multi-ethnic UK population of 909 women with gestational diabetes.
- Comprehensive follow-up over a decade via linkage to primary care records.
- The moderating effect of insulin use and levels of hyperglycaemia at diagnosis on the outcome of time to development of Type 2 diabetes was also assessed.
- Unable to study the influence of other factors related to mental health such as medication adherence and lifestyle management on the risk for Type 2 diabetes.

Manuscript word count: 2347

For peer review only - http://bmjopen.bmj.com/site/about/guidelines.xhtml

Introduction

Gestational diabetes (GDM) is defined as diabetes which occurs for the first time during pregnancy and affects between five and 10% of pregnant women.[1 2] The common mental disorders (CMD) of anxiety and depression are also highly prevalent during pregnancy, affecting at least 10% of women.[3 4] There is now evidence to support an association between GDM and antenatal (during pregnancy) depression,[5] meaning that the two conditions may be frequently co-morbid. Both conditions are associated with adverse obstetric, neonatal and longer-term outcomes for women and their children.[4 6]

Up to 60% of women with GDM will develop Type 2 diabetes at some point in their lifetime following pregnancy.[7] This is thought to be due to the pathological process of insulin resistance common to both GDM and Type 2 diabetes. Pregnancy itself is a state of insulin resistance. While this may resolve following pregnancy, there is a risk of advancing insulin resistance in some women who have experienced GDM as they age. Other risk factors for development of Type 2 diabetes include socio-economic deprivation and non-White ethnicity.[8]

There are also clinical markers of risk during pregnancy for the future development of Type 2 diabetes. These include fasting glucose at the time of the OGTT (oral glucose tolerance test): the diagnostic test for GDM.[8] Yet there is evidence that glycaemic control during pregnancy in those with GDM impacts future Type 2 diabetes risk independently of baseline fasting glucose at the time of GDM diagnosis.[9 10] There is also evidence of an increased risk in women whose GDM has required the use of insulin; this may be a marker of increased severity of insulin resistance and/or poorer glycaemic control post diagnosis.[11 12] In the non-pregnant population of those with Type 2 diabetes, hyperglycaemia was significantly associated with depression in a meta-analysis of 24 studies.[13] Indeed there is evidence for a relationship between depression and various factors known to impact glycaemic control; those with depression and Type 2 diabetes may be less likely to adhere to lifestyle interventions, such as those involving diet and physical activity[14] and also medication[15]: both of which are also elements of GDM management.[16] Thus it may be hypothesised that in women with GDM, co-morbid CMD during pregnancy could increase the risk of Type 2 diabetes in the future.

Despite this, none of the recent risk prediction models for the development of Type 2 diabetes following GDM have included mental disorder.[17] Greater understanding of the impact of antenatal CMD on risk for subsequent Type 2 diabetes in women with GDM could provide further insights into the risk profile of women who are more likely to develop Type 2 diabetes. Knowledge of such modifiable risk factors could inform interventions to reduce this risk. The aim of this study was to compare, in a population of women with GDM, the time to development of Type 2 diabetes in those experiencing antenatal CMD, with those not experiencing antenatal CMD.

For peer review only

Methods

Sample: women with gestational diabetes (GDM)

Born in Bradford (BiB) is a prospective longitudinal cohort of 12,450 women with 13,758 pregnancies in Bradford. Bradford is a city in the north of England and has high levels of deprivation; 60% of babies born in Bradford are born into the poorest 20% of the English and Welsh population according to Index of Multiple Deprivation (IMD 2010). Its ethnic makeup is predominantly bi-ethnic: Pakistani and White British.

Pregnant women were recruited to the BiB cohort between 2007 and 2010,[18] when attending a routine appointment for a two hour 75g OGTT, as currently recommended by the UK's National Institute for Health and Care Excellence (NICE) as the gold standard for diagnosing GDM.[16] It is offered to all women in Bradford, usually between 26 and 28 weeks gestation. Consent was obtained for record linkage to maternity and primary care records via SystmOne: a clinical computer system used by almost all general practices in Bradford and which provides primary care data on diagnoses and prescriptions.

GDM was diagnosed according to modified World Health Organisation (WHO) criteria (either fasting glucose ≥6·1 mmol/l or two hour post-load glucose ≥7·8 mmol/l).[19] The sample used for analysis was women with GDM and linkage to primary care records (n=994; see Figure 1). If women had more than one GDM affected pregnancy in the cohort, the first pregnancy (and its date of delivery) was used as the start of the 'at risk' period for Type 2 diabetes. Women with unknown date of delivery also were excluded due to being unable to determine the start of their 'at risk' period. This gave a final sample of 909 women.

Fig 1: how the sample was obtained

Exposure: antenatal common mental disorders (CMD)

As an indicator of CMD, Read codes (Clinical Terms Version 3) for diagnosis and/or treatment of CMD were extracted from GP electronic health records (EHR) alongside medication prescriptions as per previously published methods.[20] These relate to ICD-10 diagnostic groups F32 and F33 depressive episodes and recurrent depressive disorders and F41 anxiety disorders.[21] The binary variable of antenatal CMD was defined as any indicator of CMD appearing in the primary care notes between the date of conception (estimated from date of last menstrual period and/or booking scan) and the date of delivery. Further information on the codes used is provided in supplementary material (S1).

Outcome: time to development of Type 2 diabetes

Read code (CTV3) for 'Type 2 diabetes mellitus': X40J5 was used as an indicator of Type 2 diabetes development. The outcome was time to the earliest recorded code in the primary care records after the date of delivery of the GDM affected pregnancy (in years).

Covariates

There were a number of variables identified as potential confounders due to their association with both GDM and CMD but not on the causal pathway from exposure to outcome.[22] These were maternal age[8 23] at the time of the OGTT and tobacco smoking during pregnancy.[24 25] CMD occurring prior to pregnancy ('preconception' CMD) was also measured using Read codes and medication prescriptions in the primary care record from the woman's birth to the date of conception of the woman's first ever pregnancy (see supplementary material S1). Also included were the sociodemographic characteristics of maternal ethnicity (as a three category variable of Pakistani, White British or other minority ethnic group) and socioeconomic status (SES);[8 25 26] rather than using index of multiple deprivation[27] to assign SES, a five category variable of maternal education was used

For peer review only - http://bmjopen.bmj.com/site/about/guidelines.xhtml

For peer review only

a proxy, as the high levels of deprivation in BiB result in a highly skewed distribution of participants across deprivation categories. It was also hypothesised that the continuous variable of fasting glucose on the OGTT and the binary variable of GDM treated with or without insulin may be effect modifiers.

Statistical analysis

Data were analysed using Stata 15.[28] Survival analysis using Cox regression was used to compare time to development of Type 2 diabetes between exposed and unexposed groups, adjusting for relevant confounders, to produce a hazard ratio (HR).[29] The time window was date of delivery of GDM pregnancy to either date of Type 2 diabetes development or date of the last EHR extraction (09/08/2017). Interaction terms for the hypothesised effect modifiers were also included in the models. Kaplan-Meier failure curves were produced showing time to development of Type 2 diabetes between women with and without antenatal CMD.

Multiple imputation by chained equations was implemented to handle missing data.[30] 20 imputations were used according to the proportion of participants with any missing data.[31] All analysis variables, including the time variable, were included in the imputation model.[32] Estimates were obtained by pooling results using Rubin's rules.[33] Complete case analyses were also conducted.

Patient and public involvement

Women with lived experience of GDM and/or mental disorder during pregnancy were consulted from study inception to completion of the analysis. They indicated that the research question was important to them. They also informed interpretation of the study's results; for example by indicating that they found the findings reassuring. These women came from the Service User Advisory Group of the Section of Women's Mental Health at King's College London and the BiB Parent Governors Group. The authors thank them for their contribution.

For peer review only

Results

Characteristics of women included in this analysis, stratified by development of Type 2 diabetes, are presented in Table 1. 59% of women with data on ethnicity were of Pakistani ethnic origin and mean maternal age was 30 years (standard deviation (SD) 5.5). 28% of women with data on education had higher than A level (secondary school) qualifications. 9% of women self-reported tobacco smoking at some point during their pregnancy. 11% had an indicator of antenatal CMD. 15% of those with available data had an indicator of preconception CMD. 18% of women with GDM developed Type 2 diabetes during the follow-up period. Pakistani ethnicity, older age and lower education were associated with the development of Type 2 diabetes.

Table 1: Characteristics of the sample (N= 909 women)

Table 1: Characteristics of the sample (N=909 women)

	Total		Type 2 diabetes		No Type 2 diabetes		p value*
	N=909	%	N=165 (18.2%)	%	N=744 (81.8%)	%	
**Ethnicity**							
Pakistani	472	51.9	102	61.8	370	49.7	
White British	198	21.8	15	9.1	183	24.6	
Other minority ethnic group	128	14.1	24	14.6	104	14	<0.001
Missing	111	12.2	24	14.6	87	11.7	
**Maternal age (years)**							
Mean (SD)	30.3 (5.5)		32.2 (5.3)		29.9 (5.5)		<0.001
Missing	41	4.5	7	4.2	34	4.6	
**Maternal education**							
Less than 5 GCSE equivalents	206	22.7	55	33.3	151	20.3	
5 GCSE equivalents	218	24.0	37	22.4	181	24.3	
A level equivalents	87	9.6	12	7.3	75	10.1	
Higher than A level	223	24.5	24	14.6	199	26.8	
Other	63	6.9	13	7.9	50	6.7	<0.001
Missing	112	12.3	24	14.6	88	11.8	
**Maternal tobacco smoking in pregnancy**							
Yes	73	8.0	15	9.1	58	7.8	
No	722	79.4	124	75.2	598	80.4	0.470
Missing	114	12.5	26	15.8	88	11.8	
**Preconception CMD**							
Yes	123	13.5	14	8.5	109	14.7	
No	710	78.1	131	79.4	579	77.8	0.056
Missing	76	8.4	20	12.1	56	7.5	
**Antenatal CMD**							
Yes	95	10.5	17	10.3	78	10.5	
No	814	89.6	148	89.7	666	89.5	0.945

**on chi square for categorical variables and t test for continuous variables comparing groups with and without Type 2 diabetes for complete cases with no missing data on exposure*

For peer review only

For peer review only - http://bmjopen.bmj.com/site/about/guidelines.xhtml

Table 2 displays the unadjusted and adjusted results of the Cox regression for Type 2 diabetes in women with and without an indicator of antenatal CMD, using imputed data. There was no evidence of an effect of antenatal CMD on the development of Type 2 diabetes following GDM on unadjusted or adjusted analyses. These findings are illustrated in Figure 2, which shows Kaplan-Meier failure curves for time to development of Type 2 diabetes between women with and without antenatal CMD. Results of complete case analyses mirrored those of the imputed results (see supplementary material S2). There was no evidence for an interaction between antenatal CMD and fasting glucose (p=0.555) or between antenatal CMD and insulin-treated GDM (p=0.680).

Table 2: Cox regression of Type 2 diabetes in women with vs without an indicator of antenatal CMD (N=909 women)

Table 2: Cox regression of Type 2 diabetes in women with vs without an indicator of antenatal CMD (N=909 women)

	Type 2 diabetes		
**Unadjusted**	**HR**	**(95% CI)**	**p-value**
**Antenatal CMD**			
Reference category= no indicator			
Antenatal CMD indicator	0.95	(0.57, 1.57)	0.839
**Adjusted***	**HR**	**(95% CI)**	**p-value**
**Antenatal CMD**			
Reference category= no indicator			
Antenatal CMD indicator	0.94	(0.56, 1.58)	0.823

*Models using imputed data *adjusted for maternal age, education, ethnicity, smoking and preconception CMD*

Fig 2: Kaplan-Meier failure curves for time to development of Type 2 diabetes (years) between women with (red line) and without (blue line) antenatal CMD

For peer review only

For peer review only - http://bmjopen.bmj.com/site/about/guidelines.xhtml

Discussion

There was no evidence in this cohort of women with GDM that antenatal CMD increased the risk of subsequent Type 2 diabetes. The proportion of women in this sample developing Type 2 diabetes was similar to that observed in a number of previous studies, which report between 10 and 20% of women with GDM developing Type 2 diabetes over similar follow-up periods of around 10 years.[34-36] There was also no evidence that fasting glucose at the time of GDM diagnosis or insulin use, as indicators of the severity of insulin resistance, modified associations between antenatal CMD and Type 2 diabetes.

This is the first study to investigate the impact of mental disorder during pregnancy in women with GDM on subsequent Type 2 diabetes risk. It is also one of the largest samples in the UK reporting Type 2 diabetes risk following GDM.[12] The sample was drawn from an urban, deprived and multi-ethnic population. While older women and women of Pakistani ethnicity were relatively over-represented in this sample compared to both the BiB cohort as a whole and the city of Bradford,[18] this is representative of those women at risk of GDM so is not unexpected.[6 16] These same demographic predictors of Type 2 diabetes and the proportions of the sample with CMD and Type 2 diabetes indicate face validity of the measures of both exposure and outcome.

Nonetheless, measures taken from primary care records are reliant upon individuals attending primary care services. Previously published papers in this cohort have discussed the ethnic differences in detection of CMD within primary care, with some evidence that women of Pakistani ethnicity may be less likely to seek help for mental ill health.[20 37] Type 2 diabetes diagnoses in primary care records may also merely reflect the likelihood of screening; a recent systematic review, albeit only using studies of US women, found that Asian women were more likely to be tested for Type 2 diabetes following GDM than White women; pooled proportion being screened was 50% in Asian and 35% in White.[38] However, if an indicator of CMD or Type 2 diabetes in primary care records merely did reflect a tendency to engage with health services, there would have been more evidence of an association between the two measures.

While the variables of fasting glucose at the time of the OGTT and insulin use were investigated as potential moderators, there were no other measures of factors also known to be affected by the presence of CMD and which may impact on Type 2 diabetes risk, such as adherence with medication and lifestyle interventions. However, while most of the evidence of the adverse influence of depression on these behaviours comes from the Type 2 diabetes population,[14] as discussed in the introduction, there remains very little evidence in women with GDM that mental health affects these behaviours. For example, a study of 400 women with GDM in Iran found no correlation between symptoms of anxiety and depression and 'self-care behaviours' such as dietary monitoring, physical activity and blood glucose monitoring.[39] Moreover, dietary and physical activity interventions trialled in obese pregnant women (albeit prior to the development of GDM) have not found antenatal depression to be a significant barrier to adherence.[40 41] Similarly, there are factors occurring in the postnatal period which may be associated with antenatal CMD, which may mediate the risk of Type 2 diabetes and which were not explored. These include events in subsequent pregnancies such as future episodes of GDM and/or CMD, breastfeeding,[42 43] postnatal CMD[44-46]. However and changes made to diet and levels of physical activity in the postpartum which in turn may influence weight.[8]

While not the focus of this study, future research could focus on investigating the relationship between maternal mental health and these other factors known to influence metabolic risk beyond the antenatal period. Future research may also examine the impact of treatment of CMD on Type 2 diabetes risk in women with GDM, in light of some evidence that treatment of depression in those with Type 2 diabetes may improve their glycaemic control.[47 48] While it could be argued that an indicator of CMD in primary care records makes it likely that the CMD was treated, such differences

For peer review only - http://bmjopen.bmj.com/site/about/guidelines.xhtml

in outcomes between treated and untreated CMD could not be explored with the measures available in this sample. Nonetheless, the findings from this analysis that women with antenatal CMD are not at greater risk of Type 2 diabetes following GDM than women without antenatal CMD could provide reassurance to these women, particularly if replicated in other samples.

For peer review only

For peer review only - http://bmjopen.bmj.com/site/about/guidelines.xhtml

Acknowledgements

Born in Bradford is only possible because of the enthusiasm and commitment of all the children and parents in Born in Bradford. We thank all the participants, health professionals and researchers who have made Born in Bradford happen.

We gratefully acknowledge the contribution of TPP and the TPP ResearchOne team in completing study participant matching to GP primary care records and in providing ongoing informatics support.

This project is supported by the UK's National Institute for Health Research (NIHR) Applied Research Collaboration (ARC) for South London.

For peer review only

For peer review only - http://bmjopen.bmj.com/site/about/guidelines.xhtml

References

1. Behboudi-Gandevani S, Amiri M, Bidhendi Yarandi R, et al. The impact of diagnostic criteria for gestational diabetes on its prevalence: a systematic review and meta-analysis. *Diabetology & Metabolic Syndrome* 2019;11(1):11.
2. Zhu Y, Zhang C. Prevalence of gestational diabetes and risk of progression to type 2 diabetes: a global perspective. *Current diabetes reports* 2016;16(1):7.
3. Gavin NI, Gaynes BN, Lohr KN, et al. Perinatal depression: a systematic review of prevalence and incidence. *Obstetrics & Gynecology* 2005;106(5):1071-83.
4. Howard L, Molyneaux E, Dennis C, et al. Non-psychotic mental disorders in the perinatal period. *Lancet* 2014;384(9956):1775-88.
5. Wilson CA, Newham J, Rankin J, et al. Is there an increased risk of perinatal mental disorder in women with gestational diabetes? A systematic review and meta-analysis. *Diabetic Medicine* 2020;37(4):602-22.
6. Kampmann U, Madsen LR, Skajaa GO, et al. Gestational diabetes: A clinical update. *World journal of diabetes* 2015;6(8):1065-72.
7. Noctor E, Dunne FP. Type 2 diabetes after gestational diabetes: the influence of changing diagnostic criteria. *World journal of diabetes* 2015;6(2):234.
8. Di Cianni G, Lacaria E, Lencioni C, et al. Preventing type 2 diabetes and cardiovascular disease in women with gestational diabetes – The evidence and potential strategies. *Diabetes Research and Clinical Practice* 2018;145:184-92.
9. Ratner RE, Christophi CA, Metzger BE, et al. Prevention of Diabetes in Women with a History of Gestational Diabetes: Effects of Metformin and Lifestyle Interventions. *The Journal of Clinical Endocrinology & Metabolism* 2008;93(12):4774-79.
10. Yefet E, Schwartz N, Sliman B, et al. Good glycemic control of gestational diabetes mellitus is associated with the attenuation of future maternal cardiovascular risk: a retrospective cohort study. *Cardiovascular Diabetology* 2019;18(1):75.
11. Allalou A, Nalla A, Prentice KJ, et al. A Predictive Metabolic Signature for the Transition From Gestational Diabetes Mellitus to Type 2 Diabetes. *Diabetes* 2016;65(9):2529-39.
12. Rayanagoudar G, Hashi AA, Zamora J, et al. Quantification of the type 2 diabetes risk in women with gestational diabetes: a systematic review and meta-analysis of 95,750 women: Springer, 2016.
13. Lustman PJ, Anderson RJ, Freedland KE, et al. Depression and poor glycemic control: a meta-analytic review of the literature. *Diabetes Care* 2000;23(7):934-42. doi: 10.2337/diacare.23.7.934
14. Lin EH, Katon W, Von Korff M, et al. Relationship of depression and diabetes self-care, medication adherence, and preventive care. *Diabetes care* 2004;27(9):2154-60.
15. Odegard PS, Capoccia K. Medication Taking and Diabetes. *The Diabetes Educator* 2007;33(6):1014-29.
16. National Institute for Health and Care Excellence (NICE). Diabetes in pregnancy: management from preconception to the postnatal period (NG3).https://www.nice.org.uk/guidance/ng3 Accessed Jan 16, 2021
17. Lamain-de Ruiter M, Kwee A, Naaktgeboren CA, et al. Prediction models for the risk of gestational diabetes: a systematic review. *Diagnostic and prognostic research* 2017;1:3.
18. Wright J, on behalf of the Born in Bradford Scientific Collaborators Group. Cohort Profile: The Born in Bradford multi-ethnic family cohort study. *International Journal of Epidemiology* 2013;42(4):978-91.
19. Farrar D, Fairley L, Santorelli G, et al. Association between hyperglycaemia and adverse perinatal outcomes in south Asian and white British women: analysis of data from the Born in Bradford cohort. *The Lancet Diabetes & Endocrinology* 2015;3(10):795-804.

For peer review only - http://bmjopen.bmj.com/site/about/guidelines.xhtml

20. Prady SL, Pickett KE, Petherick ES, et al. Evaluation of ethnic disparities in detection of depression and anxiety in primary care during the maternal period: Combined analysis of routine and cohort data. *British Journal of Psychiatry* 2016;208(5):453-61.
21. WHO. The ICD-10 Classification of Mental and Behavioural Disorders: Clinical descriptions and diagnostic guidelines. *Geneva: World Health Organisation* 1990;https://www.who.int/classifications/icd/en/bluebook.pdf
22. VanderWeele TJ. Principles of confounder selection. *European Journal of Epidemiology* 2019;34(3):211-19.
23. McManus S, Bebbington P, Jenkins R, et al. Mental health and wellbeing in England: Adult Psychiatric Morbidity Survey 2014. *Leeds: NHS Digital* 2016;https://assets.publishing.service.gov.uk/government/uploads/system/uploads/attachment_data/file/556596/apms-2014-full-rpt.pdf.
24. Howard LM, Bekele D, Rowe M, et al. Smoking cessation in pregnant women with mental disorders: a cohort and nested qualitative study. *Bjog* 2013;120(3):362-70.
25. Bellou V, Belbasis L, Tzoulaki I, et al. Risk factors for type 2 diabetes mellitus: An exposure-wide umbrella review of meta-analyses. *PLOS ONE* 2018;13(3):e0194127.
26. Bhui K, Bhugra D, Goldberg D, et al. Cultural influences on the prevalence of common mental disorder, general practitioners' assessments and help-seeking among Punjabi and English people visiting their general practitioner. *Psychological Medicine* 2001;31(5):815-25.
27. Department for Communities and Local Government. The English Indices of Deprivation 2015. *London: UK Government* 2015;https://www.gov.uk/government/statistics/english-indices-of-deprivation-2015
28. StataCorp LP. Stata data analysis and statistical Software. *Special Edition Release* 2007;10:733.
29. Cox DR. Regression Models and Life-Tables. *Journal of the Royal Statistical Society: Series B (Methodological)* 1972;34(2):187-202. doi: 10.1111/j.2517-6161.1972.tb00899.x
30. White IR, Royston P, Wood AM. Multiple imputation using chained equations: Issues and guidance for practice. *Statistics in Medicine* 2011;30(4):377-99.
31. Bodner TE. What Improves with Increased Missing Data Imputations? *Structural Equation Modeling: A Multidisciplinary Journal* 2008;15(4):651-75.
32. White IR, Royston P. Imputing missing covariate values for the Cox model. *Statistics in medicine* 2009;28(15):1982-98.
33. Rubin DB. Multiple Imputation for Nonresponse in Surveys. *John Wiley & Sons, Inc* 1987
34. Feig DS, Zinman B, Wang X, et al. Risk of development of diabetes mellitus after diagnosis of gestational diabetes. *Canadian Medical Association Journal* 2008;179(3):229-34.
35. Grant PT, Oats JN, Beischer NA. The Long-Term Follow-up of Women with Gestational Diabetes. *Australian and New Zealand Journal of Obstetrics and Gynaecology* 1986;26(1):17-22.
36. Kim C, Newton KM, Knopp RH. Gestational Diabetes and the Incidence of Type 2 Diabetes. *A systematic review* 2002;25(10):1862-68.
37. Wilson CA, Santorelli G, Dickerson J, et al. Is there an association between anxiety and depression prior to and during pregnancy and gestational diabetes? An analysis of the Born in Bradford cohort. *Journal of Affective Disorders* 2020;276:345-50.
38. Herrick CJ, Puri R, Rahaman R, et al. Maternal Race/Ethnicity and Postpartum Diabetes Screening: A Systematic Review and Meta-Analysis. *Journal of Women's Health* 2020;29(5):609-21.
39. Kordi M, Heravan MB. The Relationship of depression, anxiety, and stress with selfcare behaviors in women with gestational diabetes. *Journal of Midwifery & Reproductive Health* 2020;8(1):2083-94.
40. Molyneaux E, Begum S, Briley AL, et al. Do elevated symptoms of depression predict adherence and outcomes in the UPBEAT randomised controlled trial of a lifestyle intervention for obese pregnant women? *BMC Pregnancy and Childbirth* 2018;18(1):378.

For peer review only - http://bmjopen.bmj.com/site/about/guidelines.xhtml

41. Bogaerts AF, Devlieger R, Nuyts E, et al. Effects of lifestyle intervention in obese pregnant women on gestational weight gain and mental health: a randomized controlled trial. *Internatonal Journal of Obesity* 2013;37:814-21.
42. Tanase-Nakao K, Arata N, Kawasaki M, et al. Potential protective effect of lactation against incidence of type 2 diabetes mellitus in women with previous gestational diabetes mellitus: A systematic review and meta-analysis. *Diabetes/Metabolism Research and Reviews* 2017;33(4):e2875.
43. Dias CC, Figueiredo B. Breastfeeding and depression: A systematic review of the literature. *Journal of Affective Disorders* 2015;171:142-54.
44. Knippen KL, Sheu J-J, Oza-Frank R, et al. Predictors of Health-Protective Behavior and Glycemia After Gestational Diabetes, NHANES 2007-2014. *Diabetes Educator* 2019;45(4):408-19.
45. Nicklas J, Miller L, Zera C, et al. Factors Associated with Depressive Symptoms in the Early Postpartum Period Among Women with Recent Gestational Diabetes Mellitus. *Maternal & Child Health Journal* 2013;17(9):1665-72.
46. Ferrari U, Banning F, Freibothe I, et al. Depressive symptoms, impaired glucose metabolism, high visceral fat, and high systolic blood pressure in a subgroup of women with recent gestational diabetes. *Journal of Psychiatric Research* 2018;97:89-93.
47. Moulton C, Hopkins C, Ismail K, et al. Repositioning of diabetes treatments for depressive symptoms: A systematic review and meta-analysis of clinical trials. *Psychoneuroendocrinology* 2018;94:91-103.
48. Petrak F, Baumeister H, Skinner TC, et al. Depression and diabetes: treatment and health-care delivery. *Lancet Diabetes & Endocrinology* 2015;3(6):472-85.

For peer review only

For peer review only - http://bmjopen.bmj.com/site/about/guidelines.xhtml

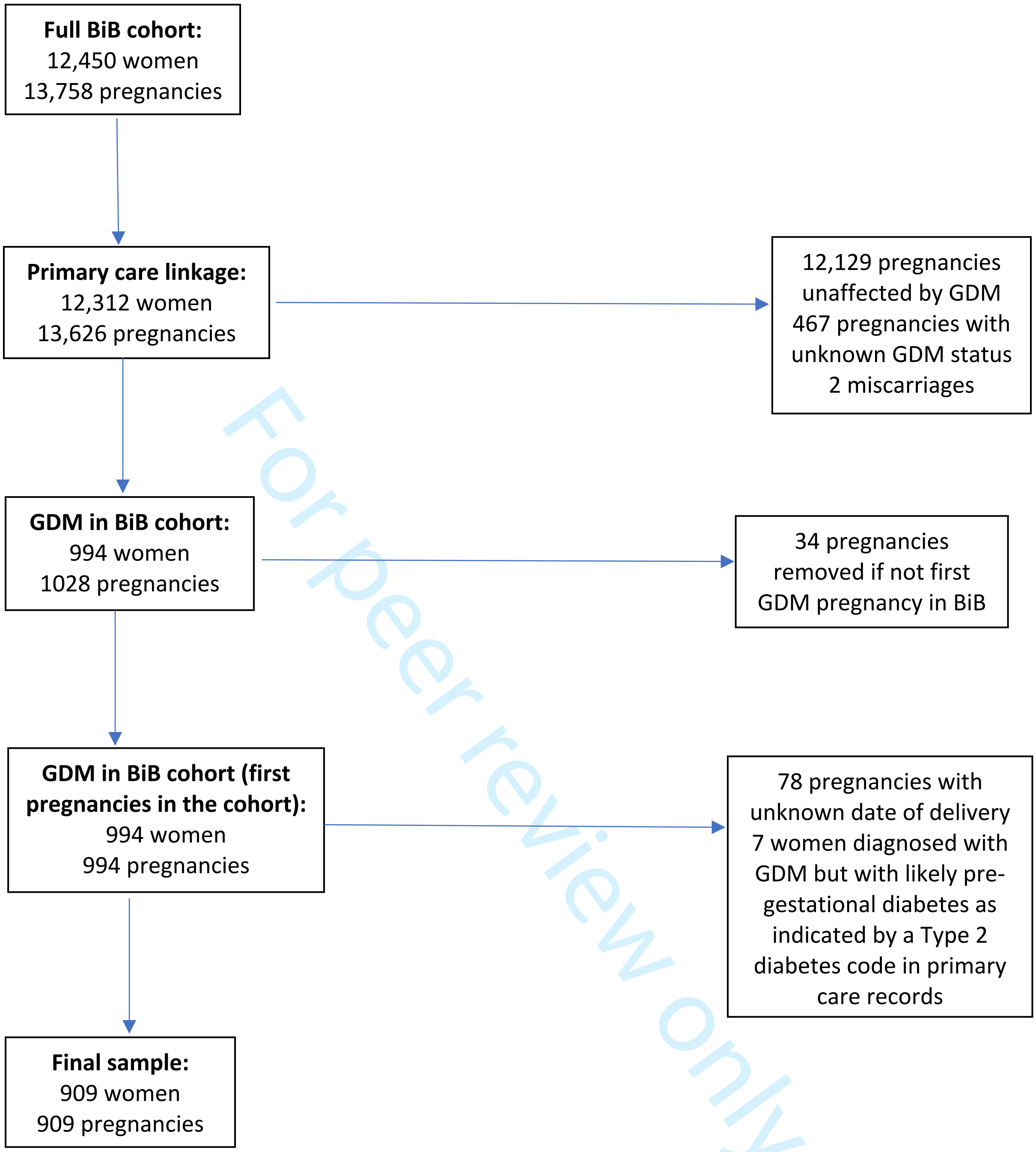

For peer review only

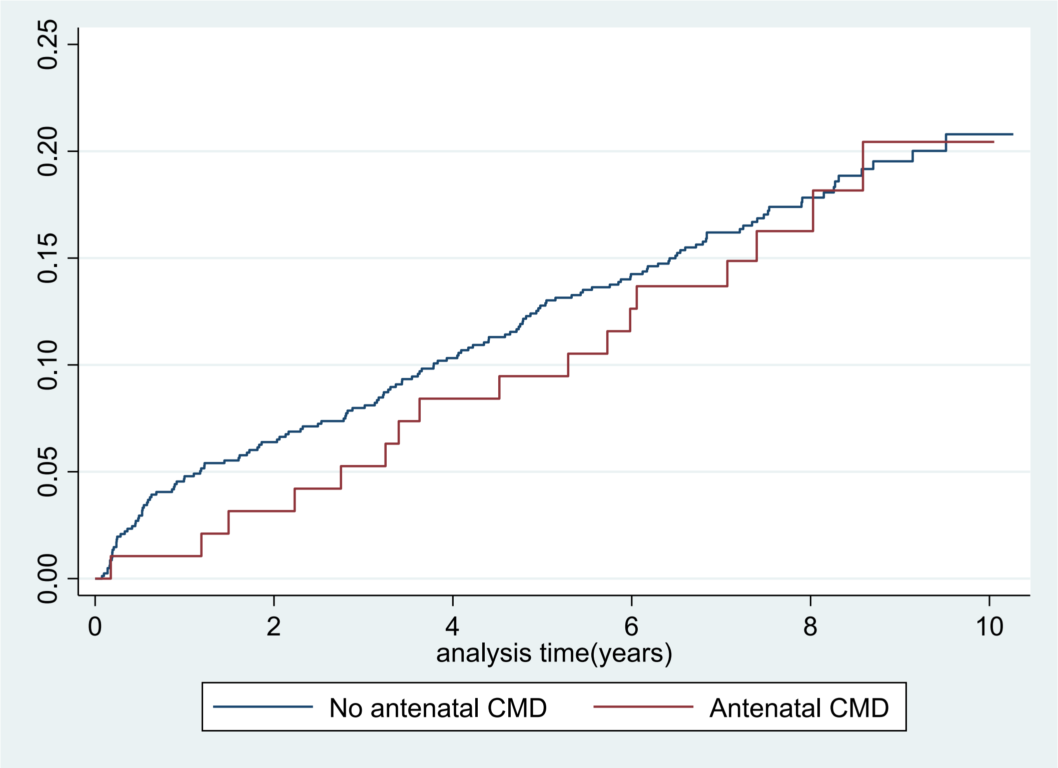
0.00
0.05
0.10
0.15
0.20
0.25
0
2
4
6
8
10
analysis time(years)
No antenatal CMD
Antenatal CMD

review only

STROBE 2007 (v4) Statement—Checklist of items that should be included in reports of *cohort studies*

Section/Topic	Item #	Recommendation	Reported on page #
**Title and abstract**	1	(*a*) Indicate the study's design with a commonly used term in the title or the abstract	3
		(*b*) Provide in the abstract an informative and balanced summary of what was done and what was found	3
**Introduction**			
Background/rationale	2	Explain the scientific background and rationale for the investigation being reported	4
Objectives	3	State specific objectives, including any prespecified hypotheses	4
**Methods**			
Study design	4	Present key elements of study design early in the paper	5
Setting	5	Describe the setting, locations, and relevant dates, including periods of recruitment, exposure, follow-up, and data collection	5
Participants	6	(*a*) Give the eligibility criteria, and the sources and methods of selection of participants. Describe methods of follow-up	5
		(*b*) For matched studies, give matching criteria and number of exposed and unexposed	N/A
Variables	7	Clearly define all outcomes, exposures, predictors, potential confounders, and effect modifiers. Give diagnostic criteria, if applicable	5
Data sources/ measurement	8*	For each variable of interest, give sources of data and details of methods of assessment (measurement). Describe comparability of assessment methods if there is more than one group	5
Bias	9	Describe any efforts to address potential sources of bias	5
Study size	10	Explain how the study size was arrived at	5 (Fig 1)
Quantitative variables	11	Explain how quantitative variables were handled in the analyses. If applicable, describe which groupings were chosen and why	6
Statistical methods	12	(*a*) Describe all statistical methods, including those used to control for confounding	6
		(*b*) Describe any methods used to examine subgroups and interactions	6
		(*c*) Explain how missing data were addressed	6
		(*d*) If applicable, explain how loss to follow-up was addressed	6
		(*e*) Describe any sensitivity analyses	6
**Results**			

For peer review only

Participants	13*	(a) Report numbers of individuals at each stage of study—eg numbers potentially eligible, examined for eligibility, confirmed eligible, included in the study, completing follow-up, and analysed	5
		(b) Give reasons for non-participation at each stage	5
		(c) Consider use of a flow diagram	5 (Fig 1)
Descriptive data	14*	(a) Give characteristics of study participants (eg demographic, clinical, social) and information on exposures and potential confounders	7 (Table 1)
		(b) Indicate number of participants with missing data for each variable of interest	7 (Table 1)
		(c) Summarise follow-up time (eg, average and total amount)	7
Outcome data	15*	Report numbers of outcome events or summary measures over time	7
Main results	16	(*a*) Give unadjusted estimates and, if applicable, confounder-adjusted estimates and their precision (eg, 95% confidence interval). Make clear which confounders were adjusted for and why they were included	8
		(*b*) Report category boundaries when continuous variables were categorized	8
		(*c*) If relevant, consider translating estimates of relative risk into absolute risk for a meaningful time period	N/A
Other analyses	17	Report other analyses done—eg analyses of subgroups and interactions, and sensitivity analyses	8
**Discussion**			
Key results	18	Summarise key results with reference to study objectives	9
**Limitations**			
Interpretation	20	Give a cautious overall interpretation of results considering objectives, limitations, multiplicity of analyses, results from similar studies, and other relevant evidence	9
Generalisability	21	Discuss the generalisability (external validity) of the study results	9
**Other information**			
Funding	22	Give the source of funding and the role of the funders for the present study and, if applicable, for the original study on which the present article is based	2

*Give information separately for cases and controls in case-control studies and, if applicable, for exposed and unexposed groups in cohort and cross-sectional studies.

Note: An Explanation and Elaboration article discusses each checklist item and gives methodological background and published examples of transparent reporting. The STROBE checklist is best used in conjunction with this article (freely available on the Web sites of PLoS Medicine at http://www.plosmedicine.org/, Annals of Internal Medicine at http://www.annals.org/, and Epidemiology at http://www.epidem.com/). Information on the STROBE Initiative is available at www.strobe-statement.org.

For peer review only

Supplementary material S1: indicators of common mental disorders (CMD) from primary care records

Prescriptions

agomelatine, alprazolam, alventa, alventa xl, angilol, ativan, bonilux, bonilux xl, buspirone hydrochloride, chloralbetaine, chloralhydrate, chloral mixture bp2000, cipralex, cipramil, circadin, citalopram, clomipramine, clomipramine hydrochloride, clonezapam, depefex, depefex xl, diazepam, dosulepin, dosulepin hydrochloride, edronax, efexor, efexor xl, escitalopram, feprapax, fluoxetine, flurazepam, fluvoxamine, fluvoxamine maleate, foraven, foraven xl, gamanil, imipramine, imipramine hydrochloride, isocarboxazid, lofepramine, lomont, loprazolam, lorazepam, lormetazepam, lustral, manerix, marplan, melatonin, meprobamate, mianserin, mianserin hydrochloride, mirtazapine, moclobemide, molipaxin, nardil, nitrazepam, optimax, oxactin, oxazepam, parnate, paroxetine, phenelzine, politid, politid xl, propranolol, propranolol hydrochloride, prothiaden, prozac, ranfaxine, ranfaxine xl, reboxetine, seroxat, sertraline, sonata, stilnoct, surmontil, syprol, temazepam, tifaxin, tifaxin xl, tranylcypromine, trazodone, trazodone hydrochloride, trimipramine, tryptophan, valdoxan, venaxx, venaxx xl, venlafaxine, venlafaxine m/r, vensir, vensir xl, welldorm, winfex, winfex xl, zaleplon, zimovane, zispin, zispinsoltab, zolpidem, zolpidem tartrate, zopiclone, allegron, anafranil, anafranil sr, chlordiazepoxide, chlordiazepoxide hydrochloride, clomethiazole, cymbalta, doxepin, duloxetine, rivotril, sinepin, sodium oxybate, triptafen, xyrem, yentreve, amitriptyline, amitriptyline hydrochloride, nortriptyline, promethazine, promethazine hydrochloride

Read codes

Depression:
1B17., 1B19., 1B1U., 2257., 62T1., E112., E1120, E1121, E1122, E1123, E1125, E1126, E112z, E113., E1130, E1131, E1132, E1135, E1136, E1137, E113z, E118., E11y2, E11z0, E11z1, E11zz, E204., E210., E211., E2110, E2112, E2B.., E2B0., E2B1., Eu320, Eu321, Eu322, Eu324, Eu325, Eu326, Eu327, Eu32B, Eu32y, Eu32z, Eu330, Eu331, Eu33y, Eu33z, Eu34., Eu340, Eu34y, Eu34z, Eu3y., Eu3y1, Eu3yy, Eu3z., Eu53., Eu530, X00SO, X00SR, X00SS, X00SU, X00TX, X40Dl, X40Dm, X760u, X7617, X761I, X761J, X761K, X761L, XE0re, XE0uv, XE1Xy, XE1Y0,
XE1Y1, XE1YC, XE1ZY, XE1Za, XE1Zb, XE1Zc, XE1Zd, XE1Zf, XE1Zg, XE1Zh, XE1Zi, XE1aY, XE1ae, XM0Ar, XM0CR, XM1GC, XSEGJ, XSGok, XSGol, XSGom, Xa02E, Xa0wV, Xa110, Xa17z, Xa1eL, Xa9E0, Xa9J0, Xa9K0, XaCHr, XaCHs, XaCIs, XaCIt, XaCIu, XaImU, XaJWh, XaKUk, XaPKm, XaPOv, XaX0C, XaY2C, XaAyL, XaB5v, XaB95, XaB9J

Anxiety:
1B13., 1B1V., 2258., 225J., E0300, E0310, E200., E2000, E2001, E2002, E2004, E2005, E200z, E201., E2010, E2011, E2012, E2013, E2014, E2015, E2016, E2017, E2018, E201A, E201B, E201C, E201z, E202., E2020, E2021, E2022, E2023, E2024, E2025, E2026, E2027, E2028, E2029, E202A, E202B, E202C, E202D, E202E, E202z, E203., E2030, E2031, E203z, E205., E207., E20y., E20y0, E20y1, E20y2, E20y3, E20yz, E20z., E28.., E280., E281., E282.,
E283., E2830, E2831, E283z, E284., E28z., Eu40., Eu400, Eu401, Eu402, Eu40y, Eu40z, Eu41., Eu410, Eu411, Eu41y, Eu41z, Eu42., Eu420, Eu421, Eu422, Eu42y, Eu42z, Eu515, Eu51y, Eu51z, Ub1T9, X00Sc, X00Sf, X761N, XE0rb, XE1Y7, XE1YA, XE1Ym, XE1Yn, XE1Zj, XE1aW, XE1bo, XM1MZ, Xa0XG, Xa0XH, Xa0XI, Xa0XJ, Xa0XK, Xa0XM, Xa0XN, Xa0XO, Xa0XP, Xa0XQ, Xa0XR, Xa0XX, Xa0XY, Xa0Xd, Xa18j, Xa18v, Xa19B, Xa3Xk, Xa3Ys, Xa7kB, XaEFB, XaP8d, XaX55, XaX56, XaX58

Comorbid depression and anxiety:
E2003, Eu412, Eu413, X00Sb

For peer review only

CMD treatment or referral for CMD treatment:
6655., 6659., 66590, 6779., 6G00., 8BK0., 8BM0., 8CQ.., 8CR7., 8F85., 8G..., 8G1.., 8G10., 8G100, 8G11., 8G12., 8G120, 8G121, 8G2.., 8G21., 8G2Z., 8G4.., 8G43., 8G4Z., 8G5.., 8G51., 8G5Z., 8G6.., 8G6Z., 8G7.., 8G7Z., 8G9.., 8G91., 8G9Z., 8HlB., 8HVO., 8H23., 8H230, 8H34., 8H38., 8H49., 8H7A., 8H7B., 8H7T., 8H7Z., 8HHp., 8HHq., 8HJ3., 8HK9., 8HkK., 8HM9., 9HZ.., 9N0T., 9N1M., 9N2B., 9N6h., 9NJ1., 9NJR., 9NJT., 9Ol.., Ub0qs, X71Ec, X71bp, X79sL, XE0iL, XE1Sa, XE1Sb, XSBbs, Xa8IB, Xa8IG, Xa8IJ, Xa8IP, Xa8IR, Xa8If, Xa8Ig, Xa8Ih, Xa8Ii, Xa8Ij, Xa8Ik, Xa8Is, Xa8It, Xa8Iu, Xa8Ix, Xa8J0, XaA8Z, XaA8c, XaA8d, XaA8u, XaA8v, XaA9W, XaA9g, XaABP, XaABQ, XaAKy, XaAMj, XaAMz, XaAOd, XaAOe, XaAOf, XaAOg, XaAOh, XaAQi, XaAQo, XaAS4, XaAU5, XaAUA, XaAXe, XaAZI, XaAbC, XaAbH, XaAdM, XaAel, XaAem, XaAen, XaAfJ, XaAh4, XaAiE, XaAiI, XaAkB, XaAkI, XaAkU, XaAnb, XaBHK, XaBIg, XaBJb, XaBJc, XaBT1, XaBTD, XaBtN, XaBvV, XaBvW, XaBvX, XaCFD, XaECG, XaEVq, XaI8j, XaINQ, XaINy, XaIOf, XaIOg, XaIOh, XaIOi, XaIOj, XaIOk, XaIOl, XaIOn, XaIOp, XaIOq, XaIOs, XaIOu, XaIOv, XaIOy, XaIOz, XaIP0, XaIP1, XaIP2, XaIP3, XaIPw, XaISp, XaISv, XaISw, XaISy, XaIT1, XaIT2, XaIT3, XaIT4, XaIT5, XaIT6, XaIT7, XaIT8, XaITA, XaITG, XaITH, XaITI, XaIUv, XaIUx, XaIUy, XaIUz, XaIV0, XaIV1, XaIV2, XaIV3, XaIV4, XaIV5, XaIV6, XaIW3, XaIW4, XaIW5, XaIW6, XaIWD, XaIWM, XaIWN, XaIWR, XaIWS, XaIWT, XaIWU, XaIWV, XaIWW, XaIWX, XaIWY, XaIWZ, XaIWa, XaIWb, XaIWx, XaIWy, XaIWz, XaIX0, XaIXS, XaIXT, XaIXU, XaIXV, XaIXW, XaIXX, XaIXY, XaIXZ, XaIXa, XaIXb, XaIXh, XaIXi, XaIXk, XaIXl, XaIXm, XaIXn, XaIXo, XaIXp, XaIXq, XaIXs, XaIXt, XaIXu, XaIYN, XaIkd, XaIkg, XaIku, XaIm4, XaIpA, XaItc, XaItx, XaIuR, XaIvk, XaIvp, XaIvq, XaIyU, XaJ4V, XaJ4w, XaJ4x, XaJOA, XaJON, XaJPu, XaJPz, XaJQ1, XaJQD, XaJQE, XaJQF, XaJQG, XaJQH, XaJQI, XaJQJ, XaJQR, XaJQS, XaJQT, XaJQU, XaJQV, XaJQW, XaJQX, XaJQY, XaJQZ, XaJRr, XaJWg, XaJr3, XaK1f, XaK5q, XaK5r, XaK6K, XaK70, XaK71, XaKAx,
XaKEz, XaKGq, XaKbb, XaL03, XaL0o, XaL0p, XaL0q, XaL0r, XaL0s, XaL0t, XaL0u, XaL0v, XaL0w, XaL2L, XaLBl, XaLCP, XaLCQ, XaLFL, XaLFk, XaLNF, XaLQw, XaLnp, XaLnq, XaLnr, XaLst, XaLsu, XaLsv, XaM2K, XaM7s, XaMGz, XaMJ8, XaMhM, XaN3a, XaN4b, XaN4c, XaN4d, XaN4e, XaN4f, XaN4g, XaNPL, XaNTc, XaONq, XaOOT, XaObo, XaOxM, XaP6T, XaP7x, XaPRF, XaPTT, XaPTU, XaPlZ, XaPvy, XaPvw, XaQBz, XaQC0, XaQWJ, XaQvz, XaR4n, XaR4s, XaR5D, XaWzW, XaX04, XaXEJ, XaXH8, XaXHm, XaXe3, XaXiH, XaXl2, XaY6o, XaY7i, XaYgS, XaZIW, XaZcf, ZV663, ZV673, ZV69., ZV690, ZV691, ZV692, ZV6D., ZV701, ZV702

CMD-related follow-up:
665.., 6654., 6658., 66580, 665A., 665A0, 665Z., 8A2.., 8A21., 8A2Z., 9H90., 9H91., 9H92., 9HA0., 9Ov.., 9Ov0., 9Ov1., 9Ov2., 9Ov3., 9Ov4., X74WN, XaJuG, XaJuK, XaJuT, XaJuV, XaJuW, XaK6d, XaK6e, XaK6f, XaK9p, XaKAK, XaLIb, XaMGL, XaMGN, XaMGO, XaMGP, XaMGQ, XaMGR, XaR9y, XaZ2p

CMD History:
146.., 1465., 1466., 1467., 146A., 146G., 146Z., 9HA1., Eu334, Xa41K, XaJWi, XaLG., ZV111

For peer review only

Supplementary material S2: Complete case analysis of Cox regression for Type 2 diabetes in women with vs without an indicator of antenatal CMD (N=750 women with GDM)

Complete case analysis of Cox regression for Type 2 diabetes in women with vs without an indicator of antenatal CMD (N=750 women with GDM)

	Type 2 diabetes		
**Unadjusted**	**HR**	**(95% CI)**	**p**
**Antenatal CMD**			
Reference category= no indicator			
Antenatal CMD indicator	1.12	(0.66, 1.92)	0.668
**Adjusted***	**HR**	**(95% CI)**	**p**
**Antenatal CMD**			
Reference category= no indicator			
Antenatal CMD indicator	1.12	(0.65, 1.94)	0.685

**adjusted for maternal age, education, ethnicity, smoking and preconception CMD*

For peer review only

BMJ Open

Development of Type 2 diabetes in women with co-morbid gestational diabetes and common mental disorders in the Born in Bradford cohort

Journal:	*BMJ Open*
Manuscript ID	bmjopen-2021-051498.R1
Article Type:	Original research
Date Submitted by the Author:	30-Dec-2021
Complete List of Authors:	Wilson, Claire; King's College London, Section of Women's Mental Health; South London and Maudsley NHS Foundation Trust,<br>Santorelli, Gillian; Bradford Teaching Hospitals NHS Foundation Trust<br>Reynolds, Rebecca; University of Edinburgh,<br>Simonoff, Emily; King's College London, Institute of Psychiatry, Child & Adolescent Psychiatry<br>Howard, Louise; institute of psychiatry, health service and population research<br>Ismail, Khalida; Institute of Psychiatry, Kings College London
**Primary Subject Heading**:	Epidemiology
Secondary Subject Heading:	Diabetes and endocrinology, Mental health, Obstetrics and gynaecology
Keywords:	General diabetes < DIABETES & ENDOCRINOLOGY, EPIDEMIOLOGY, Diabetes in pregnancy < DIABETES & ENDOCRINOLOGY, Maternal medicine < OBSTETRICS, Depression & mood disorders < PSYCHIATRY, PUBLIC HEALTH

SCHOLARONE™
Manuscripts

I, the Submitting Author has the right to grant and does grant on behalf of all authors of the Work (as defined in the below author licence), an exclusive licence and/or a non-exclusive licence for contributions from authors who are: i) UK Crown employees; ii) where BMJ has agreed a CC-BY licence shall apply, and/or iii) in accordance with the terms applicable for US Federal Government officers or employees acting as part of their official duties; on a worldwide, perpetual, irrevocable, royalty-free basis to BMJ Publishing Group Ltd ("BMJ") its licensees and where the relevant Journal is co-owned by BMJ to the co-owners of the Journal, to publish the Work in this journal and any other BMJ products and to exploit all rights, as set out in our licence.

The Submitting Author accepts and understands that any supply made under these terms is made by BMJ to the Submitting Author unless you are acting as an employee on behalf of your employer or a postgraduate student of an affiliated institution which is paying any applicable article publishing charge ("APC") for Open Access articles. Where the Submitting Author wishes to make the Work available on an Open Access basis (and intends to pay the relevant APC), the terms of reuse of such Open Access shall be governed by a Creative Commons licence – details of these licences and which Creative Commons licence will apply to this Work are set out in our licence referred to above.

Other than as permitted in any relevant BMJ Author's Self Archiving Policies, I confirm this Work has not been accepted for publication elsewhere, is not being considered for publication elsewhere and does not duplicate material already published. I confirm all authors consent to publication of this Work and authorise the granting of this licence.

For peer review only

Development of Type 2 diabetes in women with co-morbid gestational diabetes and common mental disorders in the Born in Bradford cohort

Claire A Wilson, Section of Women's Mental Health, PO31 King's College London, De Crespigny Park, London, SE5 8AF and South London and Maudsley NHS Foundation Trust, UK

Corresponding author e-mail: claire.1.wilson@kcl.ac.uk; tel: 07729 324100, ORCID ID: 0000-0003-2169-5115

Gillian Santorelli, Born in Bradford, Bradford Teaching Hospitals NHS Foundation Trust, Bradford, UK

Rebecca M Reynolds, Centre for Cardiovascular Science, University of Edinburgh, UK

Emily Simonoff, Department of Child and Adolescent Psychiatry, King's College London and South London and Maudsley NHS Foundation Trust, UK

Louise M Howard*, Section of Women's Mental Health, King's College London and South London and Maudsley NHS Foundation Trust, UK

Khalida Ismail*, Department of Psychological Medicine, King's College London and South London and Maudsley NHS Foundation Trust, UK

*joint senior authors

Author contributions:

CAW conceived the research question, designed the study analysis plan, analysed the data and wrote the first draft of the manuscript. GS extracted the data from primary care records, approved the data analysis plan and critically revised the draft. RMR, ES, LMH and KI approved the data analysis plan and critically revised the draft. All authors (CAW, GS, RMR, ES, LMH and KI) have approved the final version and agree to be accountable for all aspects of the work.

Funding:

Born in Bradford receives core infrastructure funding from the Wellcome Trust (WT101597M) and a joint grant from the UK Medical Research Council (MRC) and Economic and Social Science Research Council (ESRC) (MR/N024397/1). CAW carried out this work as part of a UK Medical Research Council (MRC) funded Clinical Research Training Fellowship (MR/P019293/1). RMR acknowledges the support of Tommy's and the British Heart Foundation (RE/18/5/34216). KI and LMH receive salary support from the National Institute for Health Research (NIHR) Biomedical Research Centre at South London and Maudsley NHS Foundation Trust and King's College London. LMH also receives salary support from the NIHR South London Applied Research Collaboration (ARC).

Competing interests:

None to declare.

Patient consent for publication:

Not applicable.

Ethics approval:

Ethical approval for data collection in BiB was granted by Bradford Research Ethics Committee (Ref 07/H1302/112).

Data availability statement:

Information about access to data and collaborations available at https://borninbradford.nhs.uk/research/how-to-access-data/

For peer review only

For peer review only - http://bmjopen.bmj.com/site/about/guidelines.xhtml

Abstract

Objectives: to compare, in a population of women with gestational diabetes, the time to diagnosis of Type 2 diabetes in those with and without common mental disorder (depression and/or anxiety) during pregnancy.

Design and setting: prospective study of the Born in Bradford cohort in Bradford, UK.

Participants: 909 women diagnosed with gestational diabetes between 2007 and 2010, with linkage to their primary care records until 2017. The exposed population were women with an indicator of common mental disorder during pregnancy in primary care records. The unexposed were those without an indicator.

Outcome measures: time to diagnosis of Type 2 diabetes as indicated by a diagnosis in primary care records.

Analysis: time to event analysis using Cox regression was employed. Multiple imputation by chained equations was implemented to handle missing data. Models were adjusted for maternal age, ethnicity, education, preconception common mental disorders and tobacco smoking during pregnancy.

Results: 165 women (18%) were diagnosed with Type 2 diabetes over a follow-up period of around 10 years. There was no evidence of an effect of antenatal CMD on the development of Type 2 diabetes following GDM (adjusted HR 0.95; 95% CI 0.57, 1.57).

Conclusions: women with common mental disorders were not at an increased risk of Type 2 diabetes following gestational diabetes. This is reassuring for women with these co-morbidities but requires replication in other study populations.

Strengths and limitations of this study

- First study to investigate the impact of mental disorder during pregnancy in women with gestational diabetes on subsequent Type 2 diabetes risk.
- Large cohort study of a multi-ethnic UK population of 909 women with gestational diabetes.
- Comprehensive follow-up over a decade via linkage to primary care records.
- The moderating effect of insulin use and levels of hyperglycaemia at diagnosis on the outcome of time to development of Type 2 diabetes was also assessed.
- Unable to study the influence of other factors related to mental health such as medication adherence and lifestyle management on the risk for Type 2 diabetes.

Manuscript word count: 2555

For peer review only - http://bmjopen.bmj.com/site/about/guidelines.xhtml

Introduction

Gestational diabetes (GDM) is defined as diabetes which occurs for the first time during pregnancy and affects between five and 10% of pregnant women.[1 2] The common mental disorders (CMD) of anxiety and depression are also highly prevalent during pregnancy, affecting at least 10% of women.[3 4] There is now evidence to support an association between GDM and antenatal (during pregnancy) depression,[5] meaning that the two conditions may be frequently co-morbid. Both conditions are associated with adverse obstetric, neonatal and longer-term outcomes for women and their children.[4 6]

Up to 60% of women with GDM will develop Type 2 diabetes at some point in their lifetime following pregnancy.[7] This is thought to be due to the pathological process of insulin resistance common to both GDM and Type 2 diabetes. Pregnancy itself is a state of insulin resistance. While this may resolve following pregnancy, there is a risk of advancing insulin resistance in some women who have experienced GDM as they age. Other risk factors for development of Type 2 diabetes include socio-economic deprivation and non-White ethnicity.[8]

There are also clinical markers of risk during pregnancy for the future development of Type 2 diabetes. These include fasting glucose at the time of the OGTT (oral glucose tolerance test): the diagnostic test for GDM.[8] Yet there is evidence that glycaemic control during pregnancy in those with GDM impacts future Type 2 diabetes risk independently of baseline fasting glucose at the time of GDM diagnosis.[9 10] There is also evidence of an increased risk in women whose GDM has required the use of insulin; this may be a marker of increased severity of insulin resistance, relative pancreatic insufficiency and/or poorer glycaemic control post diagnosis.[11 12] In the non-pregnant population of those with Type 2 diabetes, hyperglycaemia was significantly associated with depression in a meta-analysis of 24 studies.[13] Indeed there is evidence for a relationship between depression and various factors known to impact glycaemic control; those with depression and Type 2 diabetes may be less likely to adhere to lifestyle interventions, such as those involving diet and physical activity[14] and also medication[15]: both of which are also elements of GDM management.[16] Thus it may be hypothesised that in women with GDM, co-morbid CMD during pregnancy could increase the risk of Type 2 diabetes in the future due to poorer glycaemic control during pregnancy.

Despite this, none of the recent risk prediction models for the development of Type 2 diabetes following GDM have included mental disorder.[17] Greater understanding of the impact of antenatal CMD on risk for subsequent Type 2 diabetes in women with GDM could provide further insights into the risk profile of women who are more likely to develop Type 2 diabetes. Knowledge of such modifiable risk factors could inform interventions to reduce this risk. The aim of this study was to compare, in a population of women with GDM, the time to development of Type 2 diabetes in those experiencing antenatal CMD, with those not experiencing antenatal CMD.

Methods

Sample: women with gestational diabetes (GDM)

Born in Bradford (BiB) is a prospective longitudinal cohort of 12,450 women with 13,758 pregnancies in Bradford. Bradford is a city in the north of England and has high levels of deprivation; 60% of babies born in Bradford are born into the poorest 20% of the English and Welsh population according to Index of Multiple Deprivation (IMD 2010). Its ethnic makeup is predominantly bi-ethnic: Pakistani and White British.

Pregnant women were recruited to the BiB cohort between 2007 and 2010,[18] when attending a routine appointment for a two hour 75g OGTT, as currently recommended by the UK's National Institute for Health and Care Excellence (NICE) as the gold standard for diagnosing GDM.[16] It is

For peer review only - http://bmjopen.bmj.com/site/about/guidelines.xhtml

offered to all women in Bradford, usually between 26 and 28 weeks gestation. Consent was obtained for record linkage to maternity and primary care records via SystmOne: a clinical computer system used by almost all general practices in Bradford and which provides primary care data on diagnoses and prescriptions.

GDM was diagnosed according to modified World Health Organisation (WHO) criteria (either fasting glucose ≥6·1 mmol/l or two hour post-load glucose ≥7·8 mmol/l).[19] The sample used for analysis was women with GDM and linkage to primary care records (n=994; see Figure 1). If women had more than one GDM affected pregnancy in the cohort, the first pregnancy (and its date of delivery) was used as the start of the 'at risk' period for Type 2 diabetes. Women with unknown date of delivery also were excluded due to being unable to determine the start of their 'at risk' period. This gave a final sample of 909 women.

Fig 1: how the sample was obtained

Exposure: antenatal common mental disorders (CMD)

As an indicator of CMD, Read codes (Clinical Terms Version 3) for diagnosis and/or treatment of CMD were extracted from GP electronic health records (EHR) alongside medication prescriptions as per previously published methods.[20] These relate to ICD-10 diagnostic groups F32 and F33 depressive episodes and recurrent depressive disorders and F41 anxiety disorders.[21] The binary variable of antenatal CMD was defined as any indicator of CMD appearing in the primary care notes between the estimated date of conception (estimated from date of last menstrual period and/or booking scan) and the date of delivery. Further information on the codes used is provided in supplementary material (S1).

Outcome: time to development of Type 2 diabetes

Read code (CTV3) for 'Type 2 diabetes mellitus': X40J5 was used as an indicator of Type 2 diabetes development.[22] The outcome was time to the earliest recorded code in the primary care records after the date of delivery of the GDM affected pregnancy (in years).

Covariates

There were a number of variables identified as potential confounders due to their association with both GDM and CMD but not on the causal pathway from exposure to outcome.[23] These were maternal age[8 24] at the time of the OGTT and tobacco smoking during pregnancy.[25 26] CMD occurring prior to pregnancy ('preconception' CMD) was also measured using Read codes and medication prescriptions in the primary care record from the woman's birth to the estimated date of conception of the woman's first ever pregnancy (see supplementary material S1). Also included were the sociodemographic characteristics of maternal ethnicity (as a three category variable of Pakistani, White British or other minority ethnic group) and socioeconomic status (SES);[8 26 27] rather than using index of multiple deprivation[28] to assign SES, a five category variable of maternal education was used a proxy, as the high levels of deprivation in BiB result in a highly skewed distribution of participants across deprivation categories. It was also hypothesised that the continuous variable of fasting glucose on the OGTT and the binary variable of GDM treated with or without insulin may be effect modifiers.

Statistical analysis

Data were analysed using Stata 15.[29] Survival analysis using Cox regression was used to compare time to development of Type 2 diabetes between exposed and unexposed groups, adjusting for relevant confounders, to produce a hazard ratio (HR).[30] The time window was date of delivery of GDM pregnancy to either date of Type 2 diabetes development or date of the last EHR extraction (09/08/2017). Interaction terms for the hypothesised effect modifiers were also included in the

For peer review only

models. Kaplan-Meier failure curves were produced showing time to development of Type 2 diabetes between women with and without antenatal CMD.

Multiple imputation by chained equations was implemented to handle missing data.[31] 20 imputations were used according to the proportion of participants with any missing data.[32] All analysis variables, including the time variable, were included in the imputation model.[33] Estimates were obtained by pooling results using Rubin's rules.[34] Complete case analyses were also conducted.

A sensitivity analysis removing women with co-morbid severe mental illnesses (SMI) was also conducted. These relate to ICD-10 diagnostic groups F20-29 psychotic disorders including schizophrenia, F30-31 bipolar disorder and F60 personality disorders[21] using GP electronic health records (EHR) alongside medication prescriptions as per previously published methods.[20] Further information on the codes used is provided in supplementary material (S2).

Patient and public involvement

Women with lived experience of GDM and/or mental disorder during pregnancy were consulted from study inception to completion of the analysis. They indicated that the research question was important to them. They also informed interpretation of the study's results; for example by indicating that they found the findings reassuring. These women came from the Service User Advisory Group of the Section of Women's Mental Health at King's College London and the BiB Parent Governors Group. The authors thank them for their contribution.

Results

Characteristics of women included in this analysis, stratified by development of Type 2 diabetes, are presented in Table 1. 59% of women with data on ethnicity were of Pakistani ethnic origin and mean maternal age was 30 years (standard deviation (SD) 5.5). 28% of women with data on education had higher than A level (secondary school) qualifications. 9% of women self-reported tobacco smoking at some point during their pregnancy. 11% had an indicator of antenatal CMD. 15% of those with available data had an indicator of preconception CMD. 18% of women with GDM developed Type 2 diabetes during the follow-up period. Pakistani ethnicity, older age and lower education were associated with the development of Type 2 diabetes.

For peer review only

For peer review only - http://bmjopen.bmj.com/site/about/guidelines.xhtml

Table 1: Characteristics of the sample (N= 909 women)

Table 1: Characteristics of the sample (N=909 women)

	**Total**		**Type 2 diabetes**		**No Type 2 diabetes**		**p value***
	**N=909**	**%**	**N=165 (18.2%)**	**%**	**N=744 (81.8%)**	**%**	
**Ethnicity**							
Pakistani	472	51.9	102	61.8	370	49.7	
White British	198	21.8	15	9.1	183	24.6	
Other minority ethnic group	128	14.1	24	14.6	104	14	<0.001
Missing	111	12.2	24	14.6	87	11.7	
**Maternal age (years)**							
Mean (SD)	30.3 (5.5)		32.2 (5.3)		29.9 (5.5)		<0.001
Missing	41	4.5	7	4.2	34	4.6	
**Maternal education**							
Less than 5 GCSE equivalents	206	22.7	55	33.3	151	20.3	
5 GCSE equivalents	218	24.0	37	22.4	181	24.3	
A level equivalents	87	9.6	12	7.3	75	10.1	
Higher than A level	223	24.5	24	14.6	199	26.8	
Other	63	6.9	13	7.9	50	6.7	<0.001
Missing	112	12.3	24	14.6	88	11.8	
**Maternal tobacco smoking in pregnancy**							
Yes	73	8.0	15	9.1	58	7.8	
No	722	79.4	124	75.2	598	80.4	0.470
Missing	114	12.5	26	15.8	88	11.8	
**Preconception CMD**							
Yes	123	13.5	14	8.5	109	14.7	
No	710	78.1	131	79.4	579	77.8	0.056
Missing	76	8.4	20	12.1	56	7.5	
**Antenatal CMD**							
Yes	95	10.5	17	10.3	78	10.5	
No	814	89.6	148	89.7	666	89.5	0.945
**Fasting glucose (mmol/l)**							
Mean (SD)	5.2 (1.09)		6.2 (1.5)		5 (0.83)		<0.001
Missing	41	4.5	7	4.2	34	4.6	
**Insulin treated GDM**							
Yes	420	46.2	47	28.5	373	50.1	<0.001
No	489	53.8	118	71.5	371	49.9	

**on chi square for categorical variables and t test for continuous variables comparing groups with and without Type 2 diabetes for complete cases with no missing data on exposure*

For peer review only - http://bmjopen.bmj.com/site/about/guidelines.xhtml

The median time taken for 15% of women to develop Type 2 diabetes in the group unexposed to antenatal CMD was 6.5 years (95% CI 4.9, 7.9) versus 7.4 years (95% CI 3.6, 7.9) in the exposed group. Table 2 displays the unadjusted and adjusted results of the Cox regression for Type 2 diabetes in women with and without an indicator of antenatal CMD, using imputed data. There was no evidence of an effect of antenatal CMD on the development of Type 2 diabetes following GDM on unadjusted or adjusted analyses. These findings are illustrated in Figure 2, which shows Kaplan-Meier failure curves for time to development of Type 2 diabetes between women with and without antenatal CMD. Results of complete case analyses mirrored those of the imputed results (see supplementary material S3). There was no evidence for an interaction between antenatal CMD and fasting glucose (p=0.555) or between antenatal CMD and insulin-treated GDM (p=0.680). Removal from the sample of 57 women with co-morbid SMI resulted in little change to the effect estimate (unadjusted HR 0.95 (95% CI 0.55, 1.65) and adjusted HR 0.89 (95% CI 0.51, 1.56).

Table 2: Cox regression of Type 2 diabetes in women with vs without an indicator of antenatal CMD (N=909 women)

Table 2: Cox regression of Type 2 diabetes in women with vs without an indicator of antenatal CMD (N=909 women)

	Type 2 diabetes		
**Unadjusted**	**HR**	**(95% CI)**	**p-value**
**Antenatal CMD**			
Reference category= no indicator			
Antenatal CMD indicator	0.95	(0.57, 1.57)	0.839
**Adjusted***	**HR**	**(95% CI)**	**p-value**
**Antenatal CMD**			
Reference category= no indicator			
Antenatal CMD indicator	0.94	(0.56, 1.58)	0.823

*Models using imputed data *adjusted for maternal age, education, ethnicity, smoking and preconception CMD*

Fig 2: Kaplan-Meier failure curves for time to development of Type 2 diabetes (years) between women with (red line) and without (blue line) antenatal CMD

Discussion

There was no evidence in this cohort of women with GDM that antenatal CMD increased the risk of subsequent Type 2 diabetes. The proportion of women in this sample developing Type 2 diabetes was similar to that observed in a number of previous studies, which report between 10 and 20% of women with GDM developing Type 2 diabetes over similar follow-up periods of around 10 years.[35-37] There was also no evidence that fasting glucose at the time of GDM diagnosis or insulin use, as indicators of the severity of insulin resistance, modified associations between antenatal CMD and Type 2 diabetes.

This is the first study to investigate the impact of mental disorder during pregnancy in women with GDM on subsequent Type 2 diabetes risk. It is also one of the largest samples in the UK reporting Type 2 diabetes risk following GDM.[12] The sample was drawn from an urban, deprived and multi-

For peer review only

For peer review only - http://bmjopen.bmj.com/site/about/guidelines.xhtml

ethnic population. While older women and women of Pakistani ethnicity were relatively over-represented in this sample compared to both the BiB cohort as a whole and the city of Bradford,[18] this is representative of those women at risk of GDM so is not unexpected.[6 16] These same demographic predictors of Type 2 diabetes and the proportions of the sample with CMD and Type 2 diabetes indicate face validity of the measures of both exposure and outcome.

Nonetheless, measures taken from primary care records are reliant upon individuals attending primary care services. For example, a number of studies have documented the underdiagnosis of CMD in primary care,[38 39] including during the perinatal period.[40] Previously published papers in this cohort have discussed the ethnic differences in detection of CMD within primary care, with some evidence that women of Pakistani ethnicity may be less likely to seek help for mental ill health.[20 41] Type 2 diabetes diagnoses in primary care records may also merely reflect the likelihood of screening; a recent systematic review, albeit only using studies of US women, found that Asian women were more likely to be tested for Type 2 diabetes following GDM than White women; pooled proportion being screened was 50% in Asian and 35% in White.[42] However, if an indicator of CMD or Type 2 diabetes in primary care records merely did reflect a tendency to engage with health services, there would have been more evidence of an association between the two measures. Nonetheless, while in the UK it is recommended that women who have experienced GDM undergo glucose monitoring at six weeks postpartum and annually thereafter,[16] evidence suggests patchy implementation of this guidance.[43] Therefore there is possible underdiagnosis of Type 2 diabetes following GDM among women in the UK.

While the variables of fasting glucose at the time of the OGTT and insulin use were investigated as potential moderators, there were no other measures of factors also known to be affected by the presence of CMD and which may impact on Type 2 diabetes risk, such as adherence with medication and lifestyle interventions. However, while most of the evidence of the adverse influence of depression on these behaviours comes from the Type 2 diabetes population,[14] as discussed in the introduction, there remains very little evidence in women with GDM that mental health affects these behaviours. For example, a study of 400 women with GDM in Iran found no correlation between symptoms of anxiety and depression and 'self-care behaviours' such as dietary monitoring, physical activity and blood glucose monitoring.[44] Moreover, dietary and physical activity interventions trialled in obese pregnant women (albeit prior to the development of GDM) have not found antenatal depression to be a significant barrier to adherence.[45 46] Similarly, there are factors occurring in the postnatal period which may be associated with antenatal CMD, which may mediate the risk of Type 2 diabetes and which were not explored. These include events in subsequent pregnancies such as future episodes of GDM and/or CMD, breastfeeding,[47 48] postnatal CMD[49-51] and changes made to diet and levels of physical activity in the postpartum which in turn may influence weight.[8]

While not the focus of this study, future research could focus on investigating the relationship between maternal mental health and these other factors known to influence metabolic risk beyond the antenatal period. Future research may also examine the impact of treatment of CMD on Type 2 diabetes risk in women with GDM, in light of some evidence that treatment of depression in those with Type 2 diabetes may improve their glycaemic control.[52 53] While it could be argued that an indicator of CMD in primary care records makes it likely that the CMD was treated, such differences in outcomes between treated and untreated CMD could not be explored with the measures available in this sample. Nonetheless, the findings from this analysis that women with antenatal CMD are not at greater risk of Type 2 diabetes following GDM than women without antenatal CMD could provide reassurance to these women, particularly if replicated in other samples.

For peer review only - http://bmjopen.bmj.com/site/about/guidelines.xhtml

Acknowledgements

Born in Bradford is only possible because of the enthusiasm and commitment of all the children and parents in Born in Bradford. We thank all the participants, health professionals and researchers who have made Born in Bradford happen.

We gratefully acknowledge the contribution of TPP and the TPP ResearchOne team in completing study participant matching to GP primary care records and in providing ongoing informatics support.

This project is supported by the UK's National Institute for Health Research (NIHR) Applied Research Collaboration (ARC) for South London.

For peer review only

For peer review only - http://bmjopen.bmj.com/site/about/guidelines.xhtml

References

1. Behboudi-Gandevani S, Amiri M, Bidhendi Yarandi R, et al. The impact of diagnostic criteria for gestational diabetes on its prevalence: a systematic review and meta-analysis. *Diabetology & Metabolic Syndrome* 2019;11(1):11.
2. Zhu Y, Zhang C. Prevalence of gestational diabetes and risk of progression to type 2 diabetes: a global perspective. *Current diabetes reports* 2016;16(1):7.
3. Gavin NI, Gaynes BN, Lohr KN, et al. Perinatal depression: a systematic review of prevalence and incidence. *Obstetrics & Gynecology* 2005;106(5):1071-83.
4. Howard L, Molyneaux E, Dennis C, et al. Non-psychotic mental disorders in the perinatal period. *Lancet* 2014;384(9956):1775-88.
5. Wilson CA, Newham J, Rankin J, et al. Is there an increased risk of perinatal mental disorder in women with gestational diabetes? A systematic review and meta-analysis. *Diabetic Medicine* 2020;37(4):602-22.
6. Kampmann U, Madsen LR, Skajaa GO, et al. Gestational diabetes: A clinical update. *World journal of diabetes* 2015;6(8):1065-72.
7. Noctor E, Dunne FP. Type 2 diabetes after gestational diabetes: the influence of changing diagnostic criteria. *World journal of diabetes* 2015;6(2):234.
8. Di Cianni G, Lacaria E, Lencioni C, et al. Preventing type 2 diabetes and cardiovascular disease in women with gestational diabetes – The evidence and potential strategies. *Diabetes Research and Clinical Practice* 2018;145:184-92.
9. Ratner RE, Christophi CA, Metzger BE, et al. Prevention of Diabetes in Women with a History of Gestational Diabetes: Effects of Metformin and Lifestyle Interventions. *The Journal of Clinical Endocrinology & Metabolism* 2008;93(12):4774-79.
10. Yefet E, Schwartz N, Sliman B, et al. Good glycemic control of gestational diabetes mellitus is associated with the attenuation of future maternal cardiovascular risk: a retrospective cohort study. *Cardiovascular Diabetology* 2019;18(1):75.
11. Allalou A, Nalla A, Prentice KJ, et al. A Predictive Metabolic Signature for the Transition From Gestational Diabetes Mellitus to Type 2 Diabetes. *Diabetes* 2016;65(9):2529-39.
12. Rayanagoudar G, Hashi AA, Zamora J, et al. Quantification of the type 2 diabetes risk in women with gestational diabetes: a systematic review and meta-analysis of 95,750 women: Springer, 2016.
13. Lustman PJ, Anderson RJ, Freedland KE, et al. Depression and poor glycemic control: a meta-analytic review of the literature. *Diabetes Care* 2000;23(7):934-42. doi: 10.2337/diacare.23.7.934
14. Lin EH, Katon W, Von Korff M, et al. Relationship of depression and diabetes self-care, medication adherence, and preventive care. *Diabetes care* 2004;27(9):2154-60.
15. Odegard PS, Capoccia K. Medication Taking and Diabetes. *The Diabetes Educator* 2007;33(6):1014-29.
16. NICE. Diabetes in pregnancy: management from preconception to the postnatal period (NG3). 2020;https://www.nice.org.uk/guidance/ng3 Accessed Jan 16, 2021
17. Lamain-de Ruiter M, Kwee A, Naaktgeboren CA, et al. Prediction models for the risk of gestational diabetes: a systematic review. *Diagnostic and prognostic research* 2017;1:3.
18. Wright J, on behalf of the Born in Bradford Scientific Collaborators Group. Cohort Profile: The Born in Bradford multi-ethnic family cohort study. *International Journal of Epidemiology* 2013;42(4):978-91.
19. Farrar D, Fairley L, Santorelli G, et al. Association between hyperglycaemia and adverse perinatal outcomes in south Asian and white British women: analysis of data from the Born in Bradford cohort. *The Lancet Diabetes & Endocrinology* 2015;3(10):795-804.
20. Prady SL, Pickett KE, Petherick ES, et al. Evaluation of ethnic disparities in detection of depression and anxiety in primary care during the maternal period: Combined analysis of routine and cohort data. *British Journal of Psychiatry* 2016;208(5):453-61.

For peer review only - http://bmjopen.bmj.com/site/about/guidelines.xhtml

21. WHO. The ICD-10 Classification of Mental and Behavioural Disorders: Clinical descriptions and diagnostic guidelines. *Geneva: World Health Organisation* 1990;https://www.who.int/classifications/icd/en/bluebook.pdf
22. NICE. Diagnosis of Type 2 diabetes in adults. 2021;https://cks.nice.org.uk/topics/diabetes-type-2/diagnosis/diagnosis-in-adults/ Accessed 17 Dec, 2021
23. VanderWeele TJ. Principles of confounder selection. *European Journal of Epidemiology* 2019;34(3):211-19.
24. McManus S, Bebbington P, Jenkins R, et al. Mental health and wellbeing in England: Adult Psychiatric Morbidity Survey 2014. *Leeds: NHS Digital* 2016;https://assets.publishing.service.gov.uk/government/uploads/system/uploads/attachment_data/file/556596/apms-2014-full-rpt.pdf.
25. Howard LM, Bekele D, Rowe M, et al. Smoking cessation in pregnant women with mental disorders: a cohort and nested qualitative study. *Bjog* 2013;120(3):362-70.
26. Bellou V, Belbasis L, Tzoulaki I, et al. Risk factors for type 2 diabetes mellitus: An exposure-wide umbrella review of meta-analyses. *PLOS ONE* 2018;13(3):e0194127.
27. Bhui K, Bhugra D, Goldberg D, et al. Cultural influences on the prevalence of common mental disorder, general practitioners' assessments and help-seeking among Punjabi and English people visiting their general practitioner. *Psychological Medicine* 2001;31(5):815-25.
28. Department for Communities and Local Government. The English Indices of Deprivation 2015. *London: UK Government* 2015;https://www.gov.uk/government/statistics/english-indices-of-deprivation-2015
29. StataCorp LP. Stata data analysis and statistical Software. *Special Edition Release* 2007;10:733.
30. Cox DR. Regression Models and Life-Tables. *Journal of the Royal Statistical Society: Series B (Methodological)* 1972;34(2):187-202. doi: 10.1111/j.2517-6161.1972.tb00899.x
31. White IR, Royston P, Wood AM. Multiple imputation using chained equations: Issues and guidance for practice. *Statistics in Medicine* 2011;30(4):377-99.
32. Bodner TE. What Improves with Increased Missing Data Imputations? *Structural Equation Modeling: A Multidisciplinary Journal* 2008;15(4):651-75.
33. White IR, Royston P. Imputing missing covariate values for the Cox model. *Statistics in medicine* 2009;28(15):1982-98.
34. Rubin DB. Multiple Imputation for Nonresponse in Surveys. *John Wiley & Sons, Inc* 1987
35. Feig DS, Zinman B, Wang X, et al. Risk of development of diabetes mellitus after diagnosis of gestational diabetes. *Canadian Medical Association Journal* 2008;179(3):229-34.
36. Grant PT, Oats JN, Beischer NA. The Long-Term Follow-up of Women with Gestational Diabetes. *Australian and New Zealand Journal of Obstetrics and Gynaecology* 1986;26(1):17-22.
37. Kim C, Newton KM, Knopp RH. Gestational Diabetes and the Incidence of Type 2 Diabetes. *A systematic review* 2002;25(10):1862-68.
38. Cepoiu M, McCusker J, Cole MG, et al. Recognition of depression by non-psychiatric physicians--a systematic literature review and meta-analysis. *Journal of general internal medicine* 2008;23(1):25-36. doi: 10.1007/s11606-007-0428-5 [published Online First: 10/26]
39. John A, McGregor J, Fone D, et al. Case-finding for common mental disorders of anxiety and depression in primary care: an external validation of routinely collected data. *BMC Medical Informatics and Decision Making* 2016;16(1):35. doi: 10.1186/s12911-016-0274-7
40. Howard LM, Molyneaux E, Dennis C-L, et al. Non-psychotic mental disorders in the perinatal period. *The Lancet* 2014;384(9956):1775-88. doi: 10.1016/S0140-6736(14)61276-9
41. Wilson CA, Santorelli G, Dickerson J, et al. Is there an association between anxiety and depression prior to and during pregnancy and gestational diabetes? An analysis of the Born in Bradford cohort. *Journal of Affective Disorders* 2020;276:345-50.
42. Herrick CJ, Puri R, Rahaman R, et al. Maternal Race/Ethnicity and Postpartum Diabetes Screening: A Systematic Review and Meta-Analysis. *Journal of Women's Health* 2020;29(5):609-21.

For peer review only - http://bmjopen.bmj.com/site/about/guidelines.xhtml

43. Ward RJ, Fryer AA, Hanna FW, et al. Inadequate postpartum screening for type 2 diabetes in women with previous gestation diabetes mellitus: A retrospective audit of practice over 17 years. *International Journal of Clinical Practice* 2021;75(9):e14447. doi: https://doi.org/10.1111/ijcp.14447
44. Kordi M, Heravan MB. The Relationship of depression, anxiety, and stress with selfcare behaviors in women with gestational diabetes. *Journal of Midwifery & Reproductive Health* 2020;8(1):2083-94.
45. Molyneaux E, Begum S, Briley AL, et al. Do elevated symptoms of depression predict adherence and outcomes in the UPBEAT randomised controlled trial of a lifestyle intervention for obese pregnant women? *BMC Pregnancy and Childbirth* 2018;18(1):378.
46. Bogaerts AF, Devlieger R, Nuyts E, et al. Effects of lifestyle intervention in obese pregnant women on gestational weight gain and mental health: a randomized controlled trial. *Internatonal Journal of Obesity* 2013;37:814-21.
47. Tanase-Nakao K, Arata N, Kawasaki M, et al. Potential protective effect of lactation against incidence of type 2 diabetes mellitus in women with previous gestational diabetes mellitus: A systematic review and meta-analysis. *Diabetes/Metabolism Research and Reviews* 2017;33(4):e2875.
48. Dias CC, Figueiredo B. Breastfeeding and depression: A systematic review of the literature. *Journal of Affective Disorders* 2015;171:142-54.
49. Knippen KL, Sheu J-J, Oza-Frank R, et al. Predictors of Health-Protective Behavior and Glycemia After Gestational Diabetes, NHANES 2007-2014. *Diabetes Educator* 2019;45(4):408-19.
50. Nicklas J, Miller L, Zera C, et al. Factors Associated with Depressive Symptoms in the Early Postpartum Period Among Women with Recent Gestational Diabetes Mellitus. *Maternal & Child Health Journal* 2013;17(9):1665-72.
51. Ferrari U, Banning F, Freibothe I, et al. Depressive symptoms, impaired glucose metabolism, high visceral fat, and high systolic blood pressure in a subgroup of women with recent gestational diabetes. *Journal of Psychiatric Research* 2018;97:89-93.
52. Moulton C, Hopkins C, Ismail K, et al. Repositioning of diabetes treatments for depressive symptoms: A systematic review and meta-analysis of clinical trials. *Psychoneuroendocrinology* 2018;94:91-103.
53. Petrak F, Baumeister H, Skinner TC, et al. Depression and diabetes: treatment and health-care delivery. *Lancet Diabetes & Endocrinology* 2015;3(6):472-85.

For peer review only

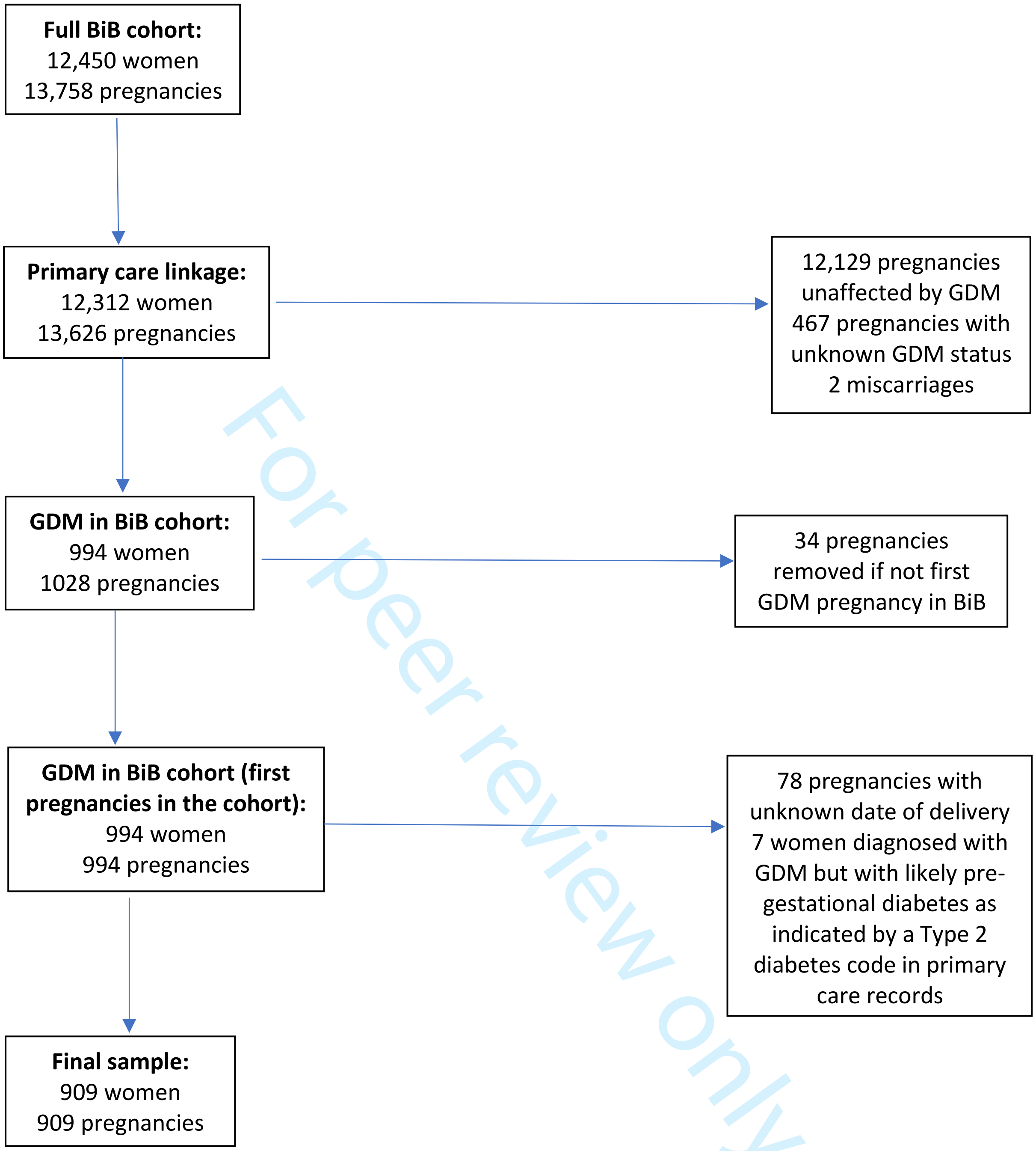

For peer review only

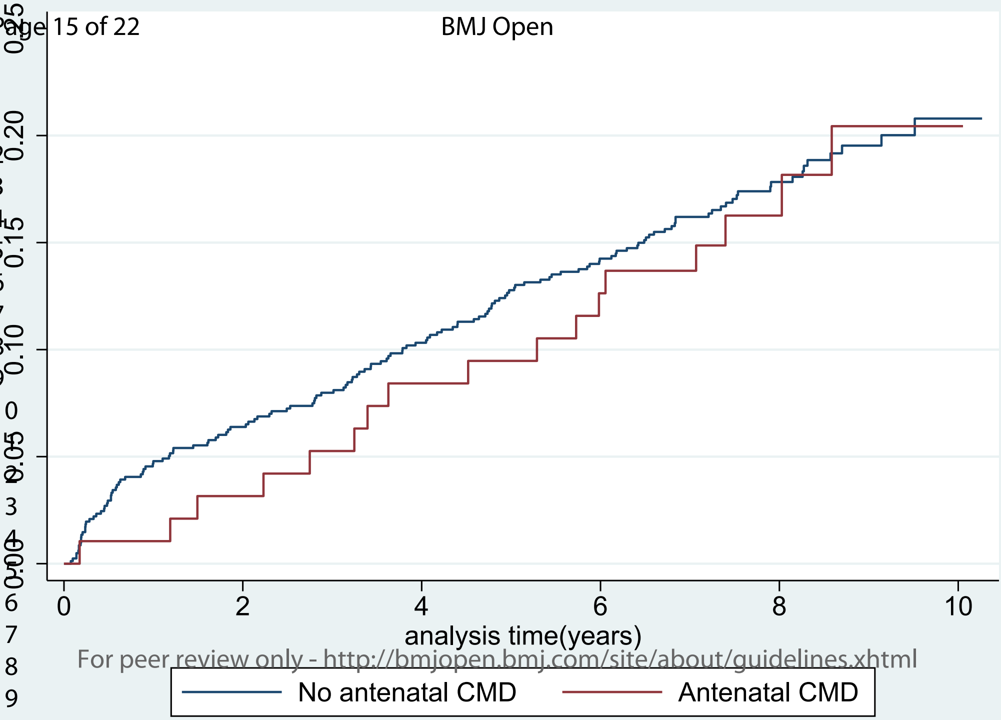

For peer review only - http://bmjopen.bmj.com/site/about/guidelines.xhtml

Supplementary material S1: indicators of common mental disorders (CMD) from primary care records

Prescriptions

agomelatine, alprazolam, alventa, alventa xl, angilol, ativan, bonilux, bonilux xl, buspirone hydrochloride, chloralbetaine, chloralhydrate, chloral mixture bp2000, cipralex, cipramil, circadin, citalopram, clomipramine, clomipramine hydrochloride, clonezapam, depefex, depefex xl, diazepam, dosulepin, dosulepin hydrochloride, edronax, efexor, efexor xl, escitalopram, feprapax, fluoxetine, flurazepam, fluvoxamine, fluvoxamine maleate, foraven, foraven xl, gamanil, imipramine, imipramine hydrochloride, isocarboxazid, lofepramine, lomont, loprazolam, lorazepam, lormetazepam, lustral, manerix, marplan, melatonin, meprobamate, mianserin, mianserin hydrochloride, mirtazapine, moclobemide, molipaxin, nardil, nitrazepam, optimax, oxactin, oxazepam, parnate, paroxetine, phenelzine, politid, politid xl, propranolol, propranolol hydrochloride, prothiaden, prozac, ranfaxine, ranfaxine xl, reboxetine, seroxat, sertraline, sonata, stilnoct, surmontil, syprol, temazepam, tifaxin, tifaxin xl, tranylcypromine, trazodone, trazodone hydrochloride, trimipramine, tryptophan, valdoxan, venaxx, venaxx xl, venlafaxine, venlafaxine m/r, vensir, vensir xl, welldorm, winfex, winfex xl, zaleplon, zimovane, zispin, zispinsoltab, zolpidem, zolpidem tartrate, zopiclone, allegron, anafranil, anafranil sr, chlordiazepoxide, chlordiazepoxide hydrochloride, clomethiazole, cymbalta, doxepin, duloxetine, rivotril, sinepin, sodium oxybate, triptafen, xyrem, yentreve, amitriptyline, amitriptyline hydrochloride, nortriptyline, promethazine, promethazine hydrochloride

Read codes

Depression:
1B17., 1B19., 1B1U., 2257., 62T1., E112., E1120, E1121, E1122, E1123, E1125, E1126, E112z, E113., E1130, E1131, E1132, E1135, E1136, E1137, E113z, E118., E11y2, E11z0, E11z1, E11zz, E204., E210., E211., E2110, E2112, E2B.., E2B0., E2B1., Eu320, Eu321, Eu322, Eu324, Eu325, Eu326, Eu327, Eu32B, Eu32y, Eu32z, Eu330, Eu331, Eu33y, Eu33z, Eu34., Eu340, Eu34y, Eu34z, Eu3y., Eu3y1, Eu3yy, Eu3z., Eu53., Eu530, X00SO, X00SR, X00SS, X00SU, X00TX, X40Dl, X40Dm, X760u, X7617, X761I, X761J, X761K, X761L, XE0re, XE0uv, XE1Xy, XE1Y0,
XE1Y1, XE1YC, XE1ZY, XE1Za, XE1Zb, XE1Zc, XE1Zd, XE1Zf, XE1Zg, XE1Zh, XE1Zi, XE1aY, XE1ae, XM0Ar, XM0CR, XM1GC, XSEGJ, XSGok, XSGol, XSGom, Xa02E, Xa0wV, Xa110, Xa17z, Xa1eL, Xa9E0, Xa9J0, Xa9K0, XaCHr, XaCHs, XaCIs, XaCIt, XaCIu, XaImU, XaJWh, XaKUk, XaPKm, XaPOv, XaX0C, XaY2C, XaAyL, XaB5v, XaB95, XaB9J

Anxiety:
1B13., 1B1V., 2258., 225J., E0300, E0310, E200., E2000, E2001, E2002, E2004, E2005, E200z, E201., E2010, E2011, E2012, E2013, E2014, E2015, E2016, E2017, E2018, E201A, E201B, E201C, E201z, E202., E2020, E2021, E2022, E2023, E2024, E2025, E2026, E2027, E2028, E2029, E202A, E202B, E202C, E202D, E202E, E202z, E203., E2030, E2031, E203z, E205., E207., E20y., E20y0, E20y1, E20y2, E20y3, E20yz, E20z., E28.., E280., E281., E282.,
E283., E2830, E2831, E283z, E284., E28z., Eu40., Eu400, Eu401, Eu402, Eu40y, Eu40z, Eu41., Eu410, Eu411, Eu41y, Eu41z, Eu42., Eu420, Eu421, Eu422, Eu42y, Eu42z, Eu515, Eu51y, Eu51z, Ub1T9, X00Sc, X00Sf, X761N, XE0rb, XE1Y7, XE1YA, XE1Ym, XE1Yn, XE1Zj, XE1aW, XE1bo, XM1MZ, Xa0XG, Xa0XH, Xa0XI, Xa0XJ, Xa0XK, Xa0XM, Xa0XN, Xa0XO, Xa0XP, Xa0XQ, Xa0XR, Xa0XX, Xa0XY, Xa0Xd, Xa18j, Xa18v, Xa19B, Xa3Xk, Xa3Ys, Xa7kB, XaEFB, XaP8d, XaX55, XaX56, XaX58

Comorbid depression and anxiety:
E2003, Eu412, Eu413, X00Sb

For peer review only

CMD treatment or referral for CMD treatment:
6655., 6659., 66590, 6779., 6G00., 8BK0., 8BM0., 8CQ.., 8CR7., 8F85., 8G..., 8G1.., 8G10., 8G100, 8G11., 8G12., 8G120, 8G121, 8G2.., 8G21., 8G2Z., 8G4.., 8G43., 8G4Z., 8G5.., 8G51., 8G5Z., 8G6.., 8G6Z., 8G7.., 8G7Z., 8G9.., 8G91., 8G9Z., 8HlB., 8HVO., 8H23., 8H230, 8H34., 8H38., 8H49., 8H7A., 8H7B., 8H7T., 8H7Z., 8HHp., 8HHq., 8HJ3., 8HK9., 8HkK., 8HM9., 9HZ.., 9N0T., 9N1M., 9N2B., 9N6h., 9NJ1., 9NJR., 9NJT., 9Ol.., Ub0qs, X71Ec, X71bp, X79sL, XE0iL, XE1Sa, XE1Sb, XSBbs, Xa8IB, Xa8IG, Xa8IJ, Xa8IP, Xa8IR, Xa8If, Xa8Ig, Xa8Ih, Xa8Ii, Xa8Ij, Xa8Ik, Xa8Is, Xa8It, Xa8Iu, Xa8Ix, Xa8J0, XaA8Z, XaA8c, XaA8d, XaA8u, XaA8v, XaA9W, XaA9g, XaABP, XaABQ, XaAKy, XaAMj, XaAMz, XaAOd, XaAOe, XaAOf, XaAOg, XaAOh, XaAQi, XaAQo, XaAS4, XaAU5, XaAUA, XaAXe, XaAZI, XaAbC, XaAbH, XaAdM, XaAel, XaAem, XaAen, XaAfJ, XaAh4, XaAiE, XaAil, XaAkB, XaAkI, XaAkU, XaAnb, XaBHK, XaBIg, XaBJb, XaBJc, XaBT1, XaBTD, XaBtN, XaBvV, XaBvW, XaBvX, XaCFD, XaECG, XaEVq, XaI8j, XaINQ, XaINy, XaIOf, XaIOg, XaIOh, XaIOi, XaIOj, XaIOk, XaIOl, XaIOn, XaIOp, XaIOq, XaIOs, XaIOu, XaIOv, XaIOy, XaIOz, XaIP0, XaIP1, XaIP2, XaIP3, XaIPw, XaISp, XaISv, XaISw, XaISy, XaIT1, XaIT2, XaIT3, XaIT4, XaIT5, XaIT6, XaIT7, XaIT8, XaITA, XaITG, XaITH, XaITI, XaIUv, XaIUx, XaIUy, XaIUz, XaIV0, XaIV1, XaIV2, XaIV3, XaIV4, XaIV5, XaIV6, XaIW3, XaIW4, XaIW5, XaIW6, XaIWD, XaIWM, XaIWN, XaIWR, XaIWS, XaIWT, XaIWU, XaIWV, XaIWW, XaIWX, XaIWY, XaIWZ, XaIWa, XaIWb, XaIWx, XaIWy, XaIWz, XaIX0, XaIXS, XaIXT, XaIXU, XaIXV, XaIXW, XaIXX, XaIXY, XaIXZ, XaIXa, XaIXb, XaIXh, XaIXi, XaIXk, XaIXl, XaIXm, XaIXn, XaIXo, XaIXp, XaIXq, XaIXs, XaIXt, XaIXu, XaIYN, XaIkd, XaIkg, XaIku, XaIm4, XaIpA, XaItc, XaItx, XaIuR, XaIvk, XaIvp, XaIvq, XaIyU, XaJ4V, XaJ4w, XaJ4x, XaJOA, XaJON, XaJPu, XaJPz, XaJQ1, XaJQD, XaJQE, XaJQF, XaJQG, XaJQH, XaJQI, XaJQJ, XaJQR, XaJQS, XaJQT, XaJQU, XaJQV, XaJQW, XaJQX, XaJQY, XaJQZ, XaJRr, XaJWg, XaJr3, XaK1f, XaK5q, XaK5r, XaK6K, XaK70, XaK71, XaKAx,
XaKEz, XaKGq, XaKbb, XaL03, XaL0o, XaL0p, XaL0q, XaL0r, XaL0s, XaL0t, XaL0u, XaL0v, XaL0w, XaL2L, XaLBl, XaLCP, XaLCQ, XaLFL, XaLFk, XaLNF, XaLQw, XaLnp, XaLnq, XaLnr, XaLst, XaLsu, XaLsv, XaM2K, XaM7s, XaMGz, XaMJ8, XaMhM, XaN3a, XaN4b, XaN4c, XaN4d, XaN4e, XaN4f, XaN4g, XaNPL, XaNTc, XaONq, XaOOT, XaObo, XaOxM, XaP6T, XaP7x, XaPRF, XaPTT, XaPTU, XaPlZ, XaPvy, XaPvw, XaQBz, XaQC0, XaQWJ, XaQvz, XaR4n, XaR4s, XaR5D, XaWzW, XaX04, XaXEJ, XaXH8, XaXHm, XaXe3, XaXiH, XaXl2, XaY6o, XaY7i, XaYgS, XaZIW, XaZcf, ZV663, ZV673, ZV69., ZV690, ZV691, ZV692, ZV6D., ZV701, ZV702

CMD-related follow-up:
665.., 6654., 6658., 66580, 665A., 665A0, 665Z., 8A2.., 8A21., 8A2Z., 9H90., 9H91., 9H92., 9HA0., 9Ov.., 9Ov0., 9Ov1., 9Ov2., 9Ov3., 9Ov4., X74WN, XaJuG, XaJuK, XaJuT, XaJuV, XaJuW, XaK6d, XaK6e, XaK6f, XaK9p, XaKAK, XaLIb, XaMGL, XaMGN, XaMGO, XaMGP, XaMGQ, XaMGR, XaR9y, XaZ2p

CMD History:
146.., 1465., 1466., 1467., 146A., 146G., 146Z., 9HA1., Eu334, Xa41K, XaJWi, XaLG., ZV111

Timing of preconception CMD

Women and all of their pregnancies were coded as affected by preconception CMD if there was attached to that woman any prescription or Read code for depression, anxiety, comorbid depression and anxiety, treatment of, follow up for or history of anxiety or depression dated prior to the date of conception of the first pregnancy; the first pregnancy for each woman may or may not have been a BiB pregnancy.
380 of 13,539 pregnancies in the sample had a missing date of conception due to unknown gestational age or date of delivery. In women with a pregnancy with a missing date of conception whose first pregnancy was a BiB pregnancy, if the date attached to the Read code or prescription was prior to 1 July 2006 (the earliest possible date of conception based on first recruitment into the

study), these women and their pregnancies were recorded as affected by preconception CMD. Otherwise the woman was recorded as missing information about preconception CMD (it would seem erroneous for such women to take the default of no preconception CMD which was used for women in which no Read or prescription codes were recorded).

Women with recorded parity exceeding zero (i.e. multiparity) or missing parity in their first BiB pregnancy (i.e. births occurring prior to entry into BiB with unknown date of conception) and with a Read or prescription code, would also have had to be coded as missing, i.e. they may have had preconception CMD, but the date of conception of their first birth is not known. However, extraction of data from maternity records pertaining to pre-BiB pregnancies provided years of pregnancies prior to the BiB study for 5611 women. If the date of the primary care code preceded the earliest pregnancy date for that mother (only year was available so the date of pregnancy was elected to be the earliest possible at 1 January), she was coded as affected by preconception CMD. For multiparous women without this information available, she was recorded as missing information about preconception CMD following the same rationale as the paragraph above.

Timing of antenatal CMD

Women were coded as experiencing antenatal CMD if there was a prescription or Read code for CMD in the mother's records between the estimated date of conception and date of delivery.

For peer review only

Supplementary material S2: indicators of severe mental illness (SMI) from primary care records

Women with a Read code or medication prescription for SMI at any point in their lifetime were coded as having SMI.

Prescriptions

abilify, acuphase, amisulpride, anquil, aripiprazole, asenapine, benperidol, camcolit, clopixol, clopixol, clozapine, clozaril, denzapine, depixol, depixol conc, depixol low volume, dolmatil, fluanxol, flupentixol, flupentixol decanoate, fluphenazine decanoate, haldol decanoate, invega, li-liquid, liskonum, lithium carbonate, lithium citrate, modecate, modecate concentrate, olanzapine, olanzapine embonate, orap, paliperidone, pericyazine, pimozide, piportil depot, pipotiazine, palmitate, priadel, promazine, promazine hydrochloride, quetiapine, risperdal, risperdal consta, risperidone, seroquel, seroquel xl, solian, sulpiride, sulpor, sycrest, xeplion, zaponex, zuclopenthixol, zuclopenthixol acetate, zuclopenthixol decanoate, zypadhera, haldol decanoate, Zyprexa, carbamazepine, chlorpromazine, chlorpromazine hydrochloride , convulex, depakote, dozic, fentazin, haldol, haloperidol, largactil, levomepromazine, nozinan, perphenazine, serenace, stelazine, tegretol trifluoperazine, valproic acid

Read codes

1B1b., 225E., 225F., 6656., 6657., 665B., 665C., 665D., 665E., 665F., 665G., 665H., 665J., 665K., 8HHs., 9H1.., 9H11., 9H12., 9H13., 9H14., 9H1Z., 9H2.., 9H21., 9H22., 9H23., 9H24., 9H25., 9H2Z., 9H3.., 9H31., 9H32., 9H33., 9H34., 9H3Z., 9H4.., 9H41., 9H42., 9H43., 9H44., 9H45., 9H4Z., 9H5.., 9H51., 9H52., 9H53., 9H54., 9H55., 9H5Z., 9H7.., 9H8.., 9Ol6., 9Ol7., E10.., E100., E1000, E1001, E1002, E1003, E1004, E1005, E100z, E101., E1010, E1011,
E1012, E1013, E1014, E1015, E101z, E102., E1020, E1021, E1022, E1023, E1024, E1025, E102z, E103., E1030, E1031, E1032, E1033, E1034, E1035, E103z, E104., E110., E1100, E1101, E1102, E1103, E1104, E1105, E1106, E110z, E111., E1110, E1111, E1112, E1113, E1114, E1115, E1116, E111z, E1124, E1133, E1134, E114., E1140, E1141, E1142, E1143, E1144, E1145, E1146, E114z, E115., E1150, E1151, E1152, E1153, E1154, E1155, E1156,
E115z, E116., E1160, E1161, E1162, E1163, E1164, E1165, E1166, E116z, E117., E1170, E1171, E1172, E1173, E1174, E1175, E1176, E117z, E11y., E11y0, E11y1, E11y3, E11yz, E11z., E12.., E120., E121., E122., E123., E12y., E12y0, E12yz, E12z., E13.., E130., E131., E132., E133., E134., E135., E13y., E13y0, E13y1, E13yz, E13z., E14.., E141., E1411, E141z, E14y., E14y1, E14yz, E14z., E1y.., E1z.., E21.., E2111, E2112, E2113, E211z, E212., E2120,
E2121, E2122, E212z, E213., E214., E2140, E2141, E214z, E215., E2150, E2151, E2152, E2153, E215z, E216., E217., E21y., E21y1, E21y2, E21y3, E21y4, E21y5, E21y6, E21y7, E21yz, E21z., Eu1.., Eu10., Eu100, Eu101, Eu102, Eu103, Eu104, Eu105, Eu106, Eu107, Eu108, Eu10y, Eu10z, Eu11., Eu110, Eu111, Eu112, Eu113, Eu114, Eu115, Eu116, Eu117, Eu11y, Eu11z, Eu12., Eu120, Eu121, Eu122, Eu123, Eu124, Eu125, Eu126, Eu127, Eu12y, Eu12z, Eu13., Eu130, Eu131, Eu132, Eu133, Eu134, Eu135, Eu136, Eu137, Eu13y, Eu13z, Eu14., Eu140, Eu141, Eu142, Eu143, Eu144, Eu145, Eu146, Eu147, Eu14y, Eu14z, Eu15., Eu150, Eu151, Eu152, Eu153, Eu154, Eu155, Eu156, Eu157, Eu15y, Eu15z, Eu16., Eu160, Eu161, Eu162, Eu163, Eu164, Eu165, Eu166, Eu167, Eu16y, Eu16z, Eu17., Eu170, Eu171, Eu172, Eu173, Eu174, Eu175, Eu176, Eu177, Eu17y, Eu17z, Eu18., Eu180, Eu181, Eu182, Eu183, Eu184, Eu185, Eu186, Eu187, Eu18y, Eu18z, Eu19., Eu190, Eu191, Eu192, Eu193, Eu194, Eu195, Eu196, Eu197, Eu19y, Eu19z, Eu1A., Eu1A0, Eu1A1, Eu1A2, Eu1A3, Eu1A4, Eu1A5, Eu1A6, Eu1A7, Eu1Ay, Eu1Az, Eu2.., Eu20., Eu200, Eu201, Eu202, Eu203, Eu204, Eu205, Eu206,

Eu20y, Eu20z, Eu21., Eu22., Eu220, Eu221, Eu222, Eu223, Eu22y, Eu22z, Eu23., Eu230, Eu231, Eu232, Eu233, Eu23y, Eu23z, Eu24., Eu25., Eu250, Eu251, Eu252, Eu25y, Eu25z, Eu26., Eu2y., Eu2z., Eu30., Eu300, Eu301, Eu302, Eu30y, Eu30z, Eu31., Eu310, Eu311, Eu312, Eu313, Eu314, Eu315, Eu316, Eu317, Eu318, Eu319, Eu31y, Eu31z, Eu323, Eu328, Eu329, Eu32A, Eu333, Eu341, Eu3z., Eu44., Eu440, Eu441, Eu442, Eu443, Eu444, Eu445, Eu446, Eu447, Eu44y, Eu44z, Eu45., Eu450, Eu451, Eu452, Eu453, Eu454, Eu455, Eu45y, Eu45z, Eu46., Eu460, Eu461, Eu46y, Eu46z, Eu5.., Eu531, Eu54., Eu55., Eu5z., Eu6.., Eu61., Eu60., Eu601, Eu602, Eu603, Eu604, Eu605, Eu606, Eu607, Eu608, Eu60y, Eu60z, Eu62., Eu620, Eu621, Eu62y, Eu62z, Ua1WW, Ub1T7, X73gl, X73gm, X73gn, X73go, X75yp, X75yv, X75yw, X75z5, X75z7, X75zA, X75zC, X75zE, X761M, X79ul, XE1Xt, XE1Xw, XE1Xx, XE1Y2, XE1Y3, XE1Y4, XE1Y5, XE1Y6, XE1YF, XE1YG, XE1YH, XE1YI, XE1YJ, XE1YK, XE1YL, XE1YM, XE1ZM, XE1ZN, XE1ZO, XE1ZP, XE1ZQ, XE1ZR, XE1ZU, XE1ZX, XE1ZZ, XE1Ze, XE1Zy, XE1aM, XE1aO, XE1aQ, XE1aS, XE1aU, XE1ag, XE1am, XE1gG, XE1ic, XE1ji, XE2RN, XE2b6, XE2b8, XE2uT, XE2un, XE2v2, XM1GG, XM1Yd, XSGon, Xa1aD, Xa1hV, Xa3WO, Xa3Xd, Xa3Xf, Xa3Xg,
Xa3Y9, Xa3aC, Xa3aF, Xa3aL, Xa3aP, Xa3aU, Xa3aV, Xa3aW, Xa3aX, Xa3at, Xa4HV, Xa4Ha, Xa8Nf, Xa8O2, Xa8OA, Xa8OE, Xa8Og, Xa8Oh, Xa8Oi, Xa8Pk, Xa8Qw, Xa8Qx, Xa8Qy, Xa8Qz, Xa9Dh, Xa9Di, Xa9Dk, Xa9Dm, Xa9Do, Xa9Dr, Xa9Ds, Xa9Dt, Xa9Du, Xa9Dv, Xa9Dw, Xa9Dx, Xa9Dy, Xa9Dz, Xa9E1, Xa9E2, Xa9EC, Xa9EE, Xa9EF, Xa9EG, Xa9EI, Xa9EM, Xa9EP, Xa9EQ, Xa9ER, Xa9EV, Xa9EW, Xa9EX, Xa9EY, Xa9EZ, Xa9Ea, Xa9Eb, Xa9Ec, Xa9Ed, Xa9Ee, Xa9Ef, Xa9Eg, Xa9Ei, Xa9Ej, Xa9Ek, Xa9El, Xa9Em, Xa9Eo, Xa9Ep, Xa9Eq, Xa9Er, Xa9Es, Xa9Et, Xa9Eu,
Xa9Ev, Xa9GR, Xa9GU, Xa9GV, Xa9GX, Xa9GZ, Xa9Gc, Xa9IW, Xa9IX, Xa9IY, Xa9IZ, Xa9Ib, Xa9Ic, Xa9Id, Xa9Ie, Xa9If, Xa9Ig, Xa9Ih, Xa9Ii, Xa9Ij, Xa9Ik, Xa9Il, Xa9Im, Xa9In, Xa9Ip, Xa9Iq, Xa9Is, Xa9Iw, Xa9Iy, Xa9J3, Xa9J4, Xa9J5, Xa9J6, Xa9JA, Xa9JC, Xa9JE, Xa9JG, Xa9JH, Xa9JJ, Xa9JK, Xa9JR, Xa9Ja, Xa9Jb, Xa9Jd, Xa9Jf, Xa9Jh, Xa9Jj, Xa9Jl, Xa9Jn, Xa9Jp, Xa9Jq, Xa9Jr, Xa9Js, Xa9Jt, Xa9Jz, Xa9K2, Xa9K3, Xa9K4, Xa9K5, XaA6j, XaA6x, XaA9j, XaA9r, XaA9s, XaBHL, XaBHM, XaBHN, XaBHO, XaBYV, XaBYW, XaBYX, XaBYY, XaBYZ, XaBhM, XaCHo, XaIOm, XaIWE, XaIWF, XaIWG, XaIWH, XaIWI, XaIWJ, XaIWK, XaIWL, XaIXj, XaJQO, XaKUl, XaKUm, XaL19, XaLIa, XaMwc, XaMwd, XaMwe, XaNlN, XaPYK, XaPYL, XaX51, XaX52, XaX53, XaX54, XaY1Y

For peer review only

Supplementary material S3: Complete case analysis of Cox regression for Type 2 diabetes in women with vs without an indicator of antenatal CMD (N=750 women with GDM)

Complete case analysis of Cox regression for Type 2 diabetes in women with vs without an indicator of antenatal CMD (N=750 women with GDM)

	Type 2 diabetes			
**Unadjusted**	**HR**	**(95% CI)**		**p**
**Antenatal CMD**				
Reference category= no indicator				
Antenatal CMD indicator	1.12	(0.66,	1.92)	0.668
**Adjusted***	**HR**	**(95% CI)**		**p**
**Antenatal CMD**				
Reference category= no indicator				
Antenatal CMD indicator	1.12	(0.65,	1.94)	0.685

**adjusted for maternal age, education, ethnicity, smoking and preconception CMD*

For peer review only

STROBE 2007 (v4) Statement—Checklist of items that should be included in reports of *cohort studies*

**Section/Topic**	**Item #**	**Recommendation**	**Reported on page #**
**Title and abstract**	1	(*a*) Indicate the study's design with a commonly used term in the title or the abstract	3
		(*b*) Provide in the abstract an informative and balanced summary of what was done and what was found	3
**Introduction**			
Background/rationale	2	Explain the scientific background and rationale for the investigation being reported	4
Objectives	3	State specific objectives, including any prespecified hypotheses	4
**Methods**			
Study design	4	Present key elements of study design early in the paper	5
Setting	5	Describe the setting, locations, and relevant dates, including periods of recruitment, exposure, follow-up, and data collection	5
Participants	6	(*a*) Give the eligibility criteria, and the sources and methods of selection of participants. Describe methods of follow-up	5
		(*b*) For matched studies, give matching criteria and number of exposed and unexposed	N/A
Variables	7	Clearly define all outcomes, exposures, predictors, potential confounders, and effect modifiers. Give diagnostic criteria, if applicable	5
Data sources/ measurement	8*	For each variable of interest, give sources of data and details of methods of assessment (measurement). Describe comparability of assessment methods if there is more than one group	5
Bias	9	Describe any efforts to address potential sources of bias	5
Study size	10	Explain how the study size was arrived at	5 (Fig 1)
Quantitative variables	11	Explain how quantitative variables were handled in the analyses. If applicable, describe which groupings were chosen and why	6
Statistical methods	12	(*a*) Describe all statistical methods, including those used to control for confounding	6
		(*b*) Describe any methods used to examine subgroups and interactions	6
		(*c*) Explain how missing data were addressed	6
		(*d*) If applicable, explain how loss to follow-up was addressed	6
		(*e*) Describe any sensitivity analyses	6
**Results**			

For peer review only

Participants	13*	(a) Report numbers of individuals at each stage of study—eg numbers potentially eligible, examined for eligibility, confirmed eligible, included in the study, completing follow-up, and analysed	5
		(b) Give reasons for non-participation at each stage	5
		(c) Consider use of a flow diagram	5 (Fig 1)
Descriptive data	14*	(a) Give characteristics of study participants (eg demographic, clinical, social) and information on exposures and potential confounders	7 (Table 1)
		(b) Indicate number of participants with missing data for each variable of interest	7 (Table 1)
		(c) Summarise follow-up time (eg, average and total amount)	7
Outcome data	15*	Report numbers of outcome events or summary measures over time	7
Main results	16	(*a*) Give unadjusted estimates and, if applicable, confounder-adjusted estimates and their precision (eg, 95% confidence interval). Make clear which confounders were adjusted for and why they were included	8
		(*b*) Report category boundaries when continuous variables were categorized	8
		(*c*) If relevant, consider translating estimates of relative risk into absolute risk for a meaningful time period	N/A
Other analyses	17	Report other analyses done—eg analyses of subgroups and interactions, and sensitivity analyses	8
**Discussion**			
Key results	18	Summarise key results with reference to study objectives	9
**Limitations**			
Interpretation	20	Give a cautious overall interpretation of results considering objectives, limitations, multiplicity of analyses, results from similar studies, and other relevant evidence	9
Generalisability	21	Discuss the generalisability (external validity) of the study results	9
**Other information**			
Funding	22	Give the source of funding and the role of the funders for the present study and, if applicable, for the original study on which the present article is based	2

*Give information separately for cases and controls in case-control studies and, if applicable, for exposed and unexposed groups in cohort and cross-sectional studies.

Note: An Explanation and Elaboration article discusses each checklist item and gives methodological background and published examples of transparent reporting. The STROBE checklist is best used in conjunction with this article (freely available on the Web sites of PLoS Medicine at http://www.plosmedicine.org/, Annals of Internal Medicine at http://www.annals.org/, and Epidemiology at http://www.epidem.com/). Information on the STROBE Initiative is available at www.strobe-statement.org.

For peer review only
